# Conserved temporal ordering of promoter activation implicates common mechanisms governing the immediate early response across cell types and stimuli

**DOI:** 10.1098/rsob.180011

**Published:** 2018-08-08

**Authors:** Annalaura Vacca, Masayoshi Itoh, Hideya Kawaji, Erik Arner, Timo Lassmann, Carsten O. Daub, Piero Carninci, Alistair R. R. Forrest, Yoshihide Hayashizaki, the FANTOM Consortium, Stuart Aitken, Colin A. Semple

**Affiliations:** 1 MRC Human Genetics Unit, MRC Institute of Genetics and Molecular Medicine, University of Edinburgh, Crewe Road, Edinburgh EH4 2XU, UK; 2 RIKEN Preventive Medicine and Diagnosis Innovation Program, 2F Main Research Building, 2-1 Hirosawa, Wako, Japan; 3 RIKEN Advanced Center for Computing and Communication, RIKEN Yokohama Campus, Yokohama 230-0045, Japan; 4 RIKEN Center for Life Sciences Technologies, RIKEN Yokohama Campus, Yokohama 230-0045, Japan; 5 Telethon Kids Institute, The University of Western Australia, Roberts Road, Subiaco, Western Australia, Australia; 6 Department of Biosciences and Nutrition, Karolinska Institutet, 141 86 Stockholm, Sweden; 7 Harry Perkins Institute of Medical Research, 6 Verdun Street, Nedlands, Western Australia 6009, Australia

**Keywords:** immediate early response, promoter activity, CAGE data, time series analysis

## Abstract

The promoters of immediate early genes (IEGs) are rapidly activated in response to an external stimulus. These genes, also known as primary response genes, have been identified in a range of cell types, under diverse extracellular signals and using varying experimental protocols. Whereas genomic dissection on a case-by-case basis has not resulted in a comprehensive catalogue of IEGs, a rigorous meta-analysis of eight genome-wide FANTOM5 CAGE (cap analysis of gene expression) time course datasets reveals successive waves of promoter activation in IEGs, recapitulating known relationships between cell types and stimuli: we obtain a set of 57 (42 protein-coding) candidate IEGs possessing promoters that consistently drive a rapid but transient increase in expression over time. These genes show significant enrichment for known IEGs reported previously, pathways associated with the immediate early response, and include a number of non-coding RNAs with roles in proliferation and differentiation. Surprisingly, we also find strong conservation of the ordering of activation for these genes, such that 77 pairwise promoter activation orderings are conserved. Using the leverage of comprehensive CAGE time series data across cell types, we also document the extensive alternative promoter usage by such genes, which is likely to have been a barrier to their discovery until now. The common activation ordering of the core set of early-responding genes we identify may indicate conserved underlying regulatory mechanisms. By contrast, the considerably larger number of transiently activated genes that are specific to each cell type and stimulus illustrates the breadth of the primary response.

## 1. Introduction

Human cells respond to a broad range of extracellular stimuli with a characteristic burst of transcription within minutes at many sites across the genome, known as the immediate early response (IER). The IER has been observed as an initiating event in many cellular processes, notably during differentiation, in responses to cellular stress and in inflammation. The earliest events in the IER involve the activation of the promoters of a particular set of genes, known as immediate early genes (IEGs). The promoters of IEGs are activated rapidly, and their activation is transient in normal cells [1]. However, IEGs are often dysregulated in cancers where they can become continuously activated; accordingly, some of the best-studied IEGs are known oncogenes [2]. For example, the expression of the FOS proto-oncogene normally peaks within 60 min of a stimulus and subsides after 90 min [3], in contrast with its continuous overexpression in many cancers.

IEGs possess unusually accessible promoters that allow rapid transcriptional activation in response to a stimulus without the requirement of de novo protein synthesis. Various features are thought to discriminate IEGs such as the shorter transcripts they generate and enrichments of certain transcription factor (TF) binding sites at their promoters [4]. However, current knowledge about IEGs is derived mainly from studies of individual genes or pathways, and often considers a specific cell type and stimulus. This means that comparison across studies can be confounded by experimental and technical variation, and a comprehensive catalogue of IEGs remains elusive. There is also controversy about the regulatory mechanisms governing the response of even relatively well-studied IEGs [5]. Beyond the induction of protein-coding IEG promoters, the features and underlying mechanisms of the IER are even less well understood. Some studies have implicated altered patterns of IEG splicing as playing important roles in the IER [6], while others have suggested a prominent role for lncRNA activation [7] and transcribed enhancers [8]. Approximately 20% of known IEGs are TFs, including some of the best characterized: EGR1–EGR4, FOS, FOSB, FOSL1, JUN, JUNB and MYC.

The FANTOM5 cap analysis of gene expression (CAGE) data offer a number of advantages for expression profiling because they are based upon single-molecule sequencing to avoid PCR, digestion and cloning biases. They provide up to single base-pair resolution of transcription start sites (TSSs) and promoter regions, and provide a sensitive, quantitative readout of transcriptional output accounting for the alternative promoters of each gene. The output of individual promoters is not confounded by splicing variation, and many novel lowly expressed transcripts including non-coding RNAs (ncRNAs) can be readily detected (see http://fantom.gsc.riken.jp/5/). CAGE data are thus ideally suited to studying the strong burst of transcription at promoters seen in IERs. FANTOM5 data include eight CAGE time course datasets employing unusually dense sampling at time points within 300 min of stimulation, for a variety of stimuli treating a variety of cell types. These heterogeneous datasets, produced using a common experimental platform, should be fertile ground for novel insights into the IER, but a comprehensive meta-analysis has not been performed until now.

Many previous approaches to time series analysis of expression data have been based upon differential expression between successive time points, or have clustered genes according to the similarity of their expression profiles over time [9]. Both of these approaches present problems for the analysis of CAGE data. Differential expression between time points provides poor sensitivity for lowly expressed transcripts (possessing too few reads to generate significant differences in expression), and presents serious difficulties when comparing expression profiles from datasets with somewhat different sampling points over time. Clustering approaches often rely upon arbitrary thresholds (e.g. based upon cluster size or significant enrichment of functional annotation terms) and, by definition, will miss transcripts that cannot be assigned to a cluster but may nevertheless show dynamics of interest. Hence, we refine a previously successful Bayesian model selection algorithm to classify promoter responses to pre-defined mathematical models [7].

Here, we perform extensive meta-analyses of promoter activity in the human IER, encompassing unusually diverse cell types and stimuli, to rigorously classify IEGs and estimate the core IEG repertoire active across cellular responses. We show that computational classification of the temporal activity patterns of promoters provides a potent basis for meta-analyses across time courses, exposing the combined activity of known IEGs and compelling new IEG candidates in the IEG core repertoire. We also show that the timing of the peak expression of a core set of transiently activated genes has a conserved order. This surprising outcome indicates a previously unidentified regulatory mechanism that is shared among cell types and common to diverse stimuli.

## 2. Results

**Figure 1.**
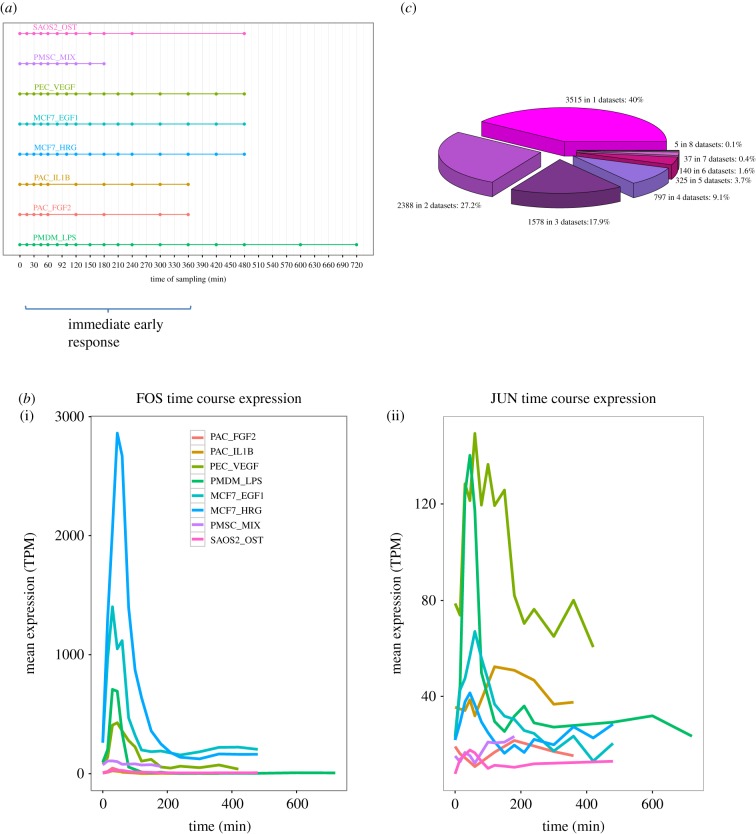
Time course datasets demonstrating the immediate early response. (*a*) Schematic of the eight time course datasets considered. Horizontal lines indicate the time span and symbols show the sampling times. Time zero corresponds to inactivated or quiescent cells in all cases. (*b*) The time course expression profile of FOS (i) and JUN (ii) in all eight datasets. Cage cluster expression (mean TPM of three replicates) is plotted against time. (*c*) The extent to which the classification of a TSS as a peak is unique to one dataset (3515 TSS) or shared between two or more datasets.

We considered eight densely sampled, and well-replicated, FANTOM5 CAGE time course datasets obtained following diverse stimuli: calcification in an osteosarcoma cell line in response to osteocalcin (SAOS2_OST), differentiation of adipose-derived primary mesenchymal stem cells in response to a drug mixture (3-isobutyl-1-methylxanthine, dexamethasone and rosiglitazone) (PMSC_MIX), differentiation of primary lymphatic endothelial cells in response to VEGF (PEC_VEGF), MCF7 breast cancer cell line responses to EGF1 (MCF7_EGF1) and to HRG (MCF7_HRG), primary aortic smooth muscle cells response to IL1b (PAC_IL1B) and FGF2 (PAC_FGF2), and primary monocyte-derived macrophage cells activation in response to LPS (PMDM_LPS). Thus, we included a variety of primary and cell line samples, tracking responses to a range of stimuli: growth factors, hormones, drugs, pro-inflammatory cytokines and bacterial endotoxin (figure 1*a*). These diverse data provided a potent resource to discover core features of the IER conserved across cell types and stimuli. All TSSs for protein-coding transcripts were represented by conservatively selected CAGE read clusters (at least 10 TPM) following Arner *et al*. [10]. As expected, the responses of known IEGs often showed characteristic expression peaks early in the time series datasets—as exemplified by FOS and JUN—though even for these well-established IEGs, we observed substantial variation in the magnitude, timing and duration of peaks across cell types and stimuli (figure 1*b*). These observations illustrate the challenges presented in IEG detection, even when studying known IEGs using a uniform experimental platform.

Optimizing and refining the approach developed by Aitken *et al.* [7] (see Material and methods), we defined four mathematical models representing archetypical expression profiles of interest over time—peak, linear, dip and decay (electronic supplementary material, figure S1)—and assessed the fit of each model to the expression profile of each gene using nested sampling to compute the marginal likelihood, log *Z* [7]. Where sufficient evidence exists (given the variation between replicates), the algorithm returns a classification of an input transcript to a model, and also computes relevant parameters of the fitted models (e.g. time and magnitude of peak expression). These parameter estimates provide a reliable basis for comparisons across time series datasets, even with different sampling densities [7], as they are not restricted to sampling times or expression values at those times.

### 2.1. Cap analysis of gene expression time series meta-analysis reveals a core complement of transiently activated promoters

Across the eight time series datasets, we considered all CAGE clusters corresponding to the TSSs of known Ensembl [11] transcripts, encompassing between 10 513 (corresponding to 7706 Ensembl genes) and 14 376 (8951 genes) protein-coding CAGE TSSs, depending on the dataset, and between 1202 (692 genes) and 1640 (858 genes) ncRNA CAGE TSSs (electronic supplementary material, table S1). Between 15 and 42% of protein-coding CAGE TSSs, and between 15 and 33% of non-coding TSSs were confidently classified to one of the four models, depending on the dataset (electronic supplementary material, figure S2 and table S2). The remainder could not be rigorously classified to a single model and were omitted from further analysis. The peak model had the highest number of assignments in all the datasets for both protein-coding and ncRNA genes; for example, of 12 132 total Ensembl protein-coding genes tested, we found 8785 Ensembl genes (72%) to peak in at least one of the datasets. By contrast, few genes were classified to the peak model in multiple datasets, with only 42 such genes shared across at least seven datasets (figure 1*c*), underlining the high variability of transcriptional responses seen for the same promoters across time series. These 42 genes constituted our ‘robust’ set of candidate IEGs (genes, TSSs and peak times listed in electronic supplementary material, File S1). We also defined a less stringent ‘permissive’ set of 1304 candidates shared across at least four out of eight datasets.

We then explored the overlap in peaking genes outside of the robust set (electronic supplementary material, table S3) and found that, for each dataset, at least 8% of peaking genes are shared with another dataset (range 8–16%) and up to 52% of peaking genes are shared (range 19–52%). The intersections between sets of three datasets became smaller consequently. Notably, approximately 50% of peaking genes are shared between datasets where the cell type is the same (MCF7 and PAC).

**Figure 2.**
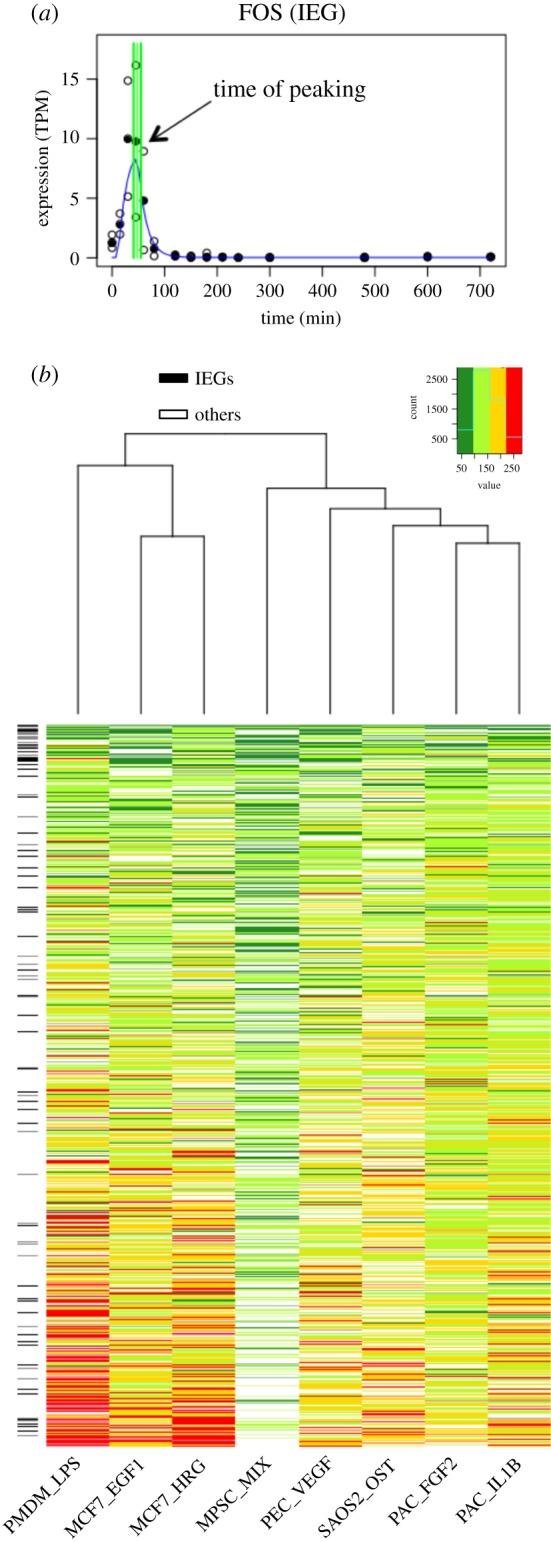
Broad trends in peak expression times across datasets. (*a*) Identification of the peak time parameter (*t*_p_) of FOS estimated from the PMDM_LPS time series (filled symbols indicate the median TPM; unfilled symbols are individual replicates; green lines represent *t*_p_ and one standard deviation above and below). (*b*) Heatmap of the times of peak TSS expression (*t*_p_) for TSSs in the permissive set for all datasets. Heatmap colours reflect the *t*_p_ for each CAGE TSS (within 100 min: dark green; 100–150 min: light green; 150–200 min: yellow; beyond 200 min: red). Known IEGs are indicated on the left by black cells.

Our model fitting approach provided parameter estimates for all promoters assigned to the same model, providing a straightforward and intuitive basis for meta-analysis. For example, comparison of the peak times (*t*_p_) (figure 2*a*) for all promoters classified as peaks in at least four datasets (the permissive set) readily demonstrated common patterns across datasets (figure 2*b*). Waves of promoter activation were evident, with certain promoters, particularly known IEGs, activated in the same early time window in multiple datasets. Hierarchical clustering of the datasets based on these peak class promoters (9% of all promoters assayed) also recapitulated known relationships between cell types and stimuli (figure 2*b*). The two datasets derived from the same breast cancer cell line (MCF7_EGF1 and MCF7_HRG) and stimulated with different ligands of the same ErbB receptor family clustered together as might be expected. We observed similar behaviour for the two primary aortic cell samples exposed to a growth factor or activated by a pro-inflammatory cytokine (PAC_FGF2 and PAC_IL1B, respectively). Thus, similarities in promoter activation dynamics (reflected in *t*_p_ parameter estimates) between datasets may reflect underlying commonalities in their underlying biology.

**Figure 3.**
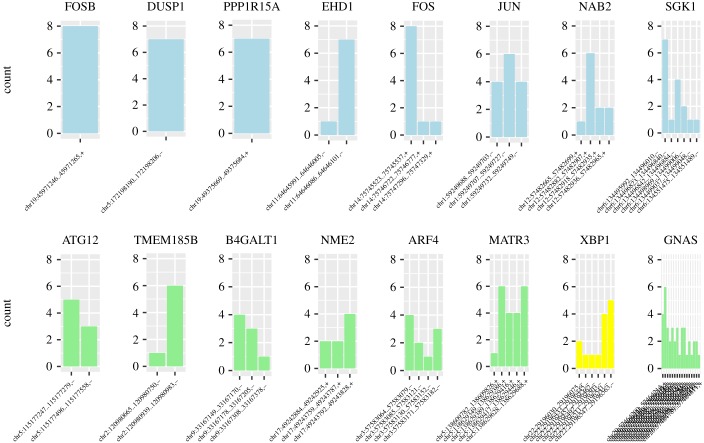
Promoter usage across time series datasets. For representative genes, bar charts show the number of datasets where each TSS peaks to illustrate the diversity of TSS usage and commonality of the peaking response. Known IEGS are shown in blue, TFs in yellow and other genes in green. FOSB has a single TSS that peaks in eight datasets, JUN has three TSS each peaking in four or more datasets and XBP1 has six TSS that peak in between one and six datasets.

The extent of alternative promoter usage across the robust set of IEGs and candidate IEGs is shown in figure 3 (see also electronic supplementary material, figure S3). Candidate IEGs show slightly greater variability in the TSSs they activate across datasets compared with known IEGs, with a greater median number TSS found to peak (3.5 compared with 2 for known IEGs). In addition, known IEGs tend to possess TSSs that are successfully classified to the peak model across a larger number of datasets (mean proportion of datasets classified as peak per TSS for known IEGs in the robust set = 4; candidate IEG mean proportion = 2.5). Thus, known IEGs tend to possess smaller numbers of alternative TSSs that also tend to show discernible peaks in the majority of the time series datasets. It is possible that these relatively stereotypical transcriptional characteristics of known IEGs may, in some cases, have led to their status as well-established IEGs. Similarly, the increased variability seen for the TSSs of candidate IEGs could have led to a failure to detect their IEG-like behaviour in former studies.

We investigated the nature of our promoter classifications by testing the enrichment of known IEGs (see Material and methods) within each class, for each dataset. The peak class was enriched for known IEGs in all datasets (electronic supplementary material, figure S4 and table S4), but failed to reach statistical significance in PMSC_MIX (OR = 1.3, *p* = 0.2). Peaking genes shared across datasets were generally associated with significant enrichments of known IEGs (table 1), with the permissive set (shared across four or more datasets) expected to contain higher numbers of false positives than the robust set (seven or more datasets). Genes possessing TSSs assigned to the peak class showed enrichments for gene ontology (GO) processes associated with transcription, cell activation, cell proliferation, cell differentiation and cancer-related terms such as cell death and apoptosis (FDR < 0.05; Material and methods) [12,13]. These terms were also consistent with previous studies of IEGs [4] as genes in the robust set showed enrichment for 285 GO terms, over 30% (88) of which were shared with the list of 773 GO terms of all known IEGs (electronic supplementary material, table S5).

**Table 1.** Enrichment of known IEGs for genes classified to the peak model in multiple datasets. Enrichment (expressed as odds ratios) and *p*-values for genes classified across different numbers of time series datasets.

shared datasets	IEGs enrichment						no. CAGE TSSs (median)
	no. genes	no. IEGs	no. CAGE TSSs (across eight datasets)	no. IEG CAGE TSSs (across eight datasets)	OR	*p*-value	
1–8 (all peaking genes)	8785	204	102 496	913	—	—	1
2–8	5270	171	71 384	853	6.3	2.2 × 10^−16^	1
3–8	2882	128	45 360	751	5.9	2.2 × 10^−16^	2
4–8	1304	86	24 616	590	5.9	2.2 × 10^−16^	2
5–8	507	56	11 528	433	7.4	2.2 × 10^−16^	3
6–8	182	35	4896	299	10.3	2.2 × 10^−16^	3
7–8	42	13	1376	124	12.6	2.2 × 10^−16^	4
8	5	2	264	18	8.3	4.6 × 10^−11^	5

### 2.2. Novel non-coding RNA candidates in the immediate early response

We next applied our classification to promoters driving the expression of non-coding transcripts and found peak promoters driving the expression of 20 ncRNA genes (across at least seven datasets), constituting the robust set of ncRNA candidate IEGs. These included promoters associated with the cellular splicing machinery, such as small nuclear RNA multi-gene families (U1, U2 and U4), which are part of the spliceosome, and SCARNA17, a small nuclear RNA which contributes to he post transcriptional modification of many snRNPs. Kalam *et al*. [14] have shown that macrophage infection with *Mycobacterium tuberculosis* results in the systematic perturbation in splicing patterns, and our results suggest more general roles for alternative splicing in the IER. However, multigene families, such as these small nuclear RNAs, present particular challenges for reliable sequence read mapping. Although probabilistic approaches to mapping ambiguously mapped reads were developed in FANTOM5 [10], we have chosen to conservatively remove these genes from the robust set, leaving a group of 15 non-coding genes with a median of five peaking TSS (table 2; electronic supplementary material, figures S5 and S6).

**Table 2.** Non-coding genes peaking in at least seven out of eight datasets. The short descriptions of the molecular function are from the genecard database [15].

gene ID	no. of shared datasets	description (PubMed ref.)
LINC00478 (MIR99AHG)	7	it has a role in cell proliferation and differentiation and it is considered a regulator of oncogenes in leukaemia (PMID: 25027842)
LINC00263	7	regulation of oligodendrocyte maturation (PMID: 25575711)
LINC-PINT	8	putative tumour suppressor (PMID: 24070194)
LINC00963	7	involved in the prostate cancer transition from androgen-dependent to androgen-independent and metastasis via the EGFR signalling pathway (PMID: 24691949)
LINC00476	8	uncharacterized lincRNA
LINC00674	7	uncharacterized lincRNA
STX18-AS1	7	uncharacterized lincRNA
DLEU2	7	critical host gene of the cell cycle inhibitory microRNAs miR-15a and miR-16-1 (PMID:19591824)
MiR-29A	7	the expression of the miR-29 family has antifibrotic effects in heart, kidney and other organs; miR-29s have also been shown to induce apoptosis and regulate cell differentiation (PMID: 22214600)
MiR-3654	7	involved in prostate cancer progression (PMID: 27297584)
MiR-21	7	oncogenic potential (PMID: 18548003)
AL928646	7	uncharacterized ncRNA
SCARNA17	7	scaRNA involved in the maturation of other RNA molecules (PMID: 12032087)
SNORD65	7	belongs to the small nucleolar RNAs, C/D family; involved in rRNA modification and alternative splicing (PMID: 26957605)
SNORD82	7	belongs to the small nucleolar RNAs, C/D family; involved in rRNA modification and alternative splicing (PMID: 26957605)

Three miRNAs are present in the robust set (table 2) including the oncogene miR-21 which was previously reported to show IEG-like behaviour in the PAC_FGF2, PAC_IL1B and MCF7_HRG time series [7]. Here, we find similar behaviour in the MCF7_EGF1, PEC_VEGF, PMSC_MIX and SAOS2_OST datasets. This extends previous studies reporting that the miR-21 mature transcript is upregulated on EGF treatment in MCF10A [16] and HeLa [17] cells. miR-29A has been associated with the viability and proliferation of mesenchymal stem cell and gastric cancer cells [18,19] and DLEU2 is a putative tumour suppressor gene that hosts two miRNAs, miR-15A and miR-16-1 which are known to inhibit cell proliferation and the colony-forming ability of tumour cell lines, and to induce apoptosis [20–22]. Seven lncRNAs also appear in the robust set (table 2), and among them, LINC00478 is particularly interesting, as it has already been reported to show IEG-like behaviour [7], is implicated in breast cancer and hosts an intronic cluster of miRNAs comprising let-7c, miR-99a and miR-125b [23]. Although poorly characterized, LINC00263, LINC-PINT and LINC00963 are thought to be involved in biological processes often triggered by IEGs, such as cell maturation, cell proliferation and the expression of growth factor receptors [24–27].

### 2.3. Known immediate early gene promoters show conserved temporal order of activation across datasets

**Figure 4.**
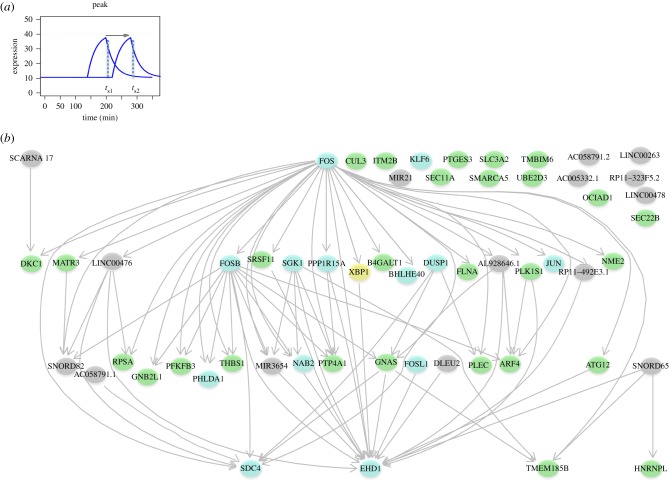
Conserved activation network. (*a*) Schematic profiles of two peaking genes, with temporal precedence indicated by the arrow. (*b*) Conserved temporal precedence between IEGs (light blue nodes), TFs (yellow nodes) ncRNA (grey nodes) and other protein-coding genes (green nodes) is shown by directed edges. A subset of IEGs in this network are also TFs (FOS, KLF6, FOSB, BHLHE40, JUN and FOSL1).

Having established common patterns of peak gene induction at similar times across datasets (figure 2*b*), we hypothesized that IEGs may also be induced in a conserved order over time. To our knowledge, the extent of conserved ordering in gene induction is unstudied in general, and in the IER, it is of particular interest for two main reasons. First, the presence of conserved gene orderings, in addition to common gene classifications, provides an additional test for functional similarity between datasets. Second, strongly conserved ordering may suggest the existence of conserved regulatory mechanisms governing the induction of these genes. To analyse the relative order of activation across the eight datasets, we compared the peak time of each gene to that of all others in the peak class. If the relative temporal order of two genes was conserved in at least seven of the eight datasets, the ordering for this pair was considered conserved and represented by an edge in the conserved activation network (figure 4).

We found 77 pairs of genes showing conserved ordering in their activation, involving 40 of the 57 genes in the robust set. FOS was the first gene to be activated (lacking a predecessor in the ordering) and SDC4, EHD1 and TMEM185B were the last. The number of conserved temporal connections observed overall is statistically significant (*p* < 5 × 10^−3^) by comparison with the distribution of expected connections, given 1 000 000 permuted datasets (Material and methods). This appears to reflect a conserved coordination in promoter activation during the IER and further supports the candidacy of the novel IEGs detected. Many genes in this network are known to participate in well-studied pathways active in the IER such as the MAPK signalling pathway as we now discuss.

### 2.4. Known immediate early genes and candidate immediate early genes participate in common signalling pathways

Having shown that the peak model described the behaviour of known IEGs, we speculated that the other genes assigned to this model might include novel candidate IEGs. Of the 42 genes in the robust set, more than two-thirds (29 genes) are not known to be IEGs and can therefore be considered to be candidate novel IEGs (henceforth candidate IEGs). Pathway analysis [28] recovers many known relationships among known IEGs as expected, centred on heavily studied IEGs such as FOS and JUN. However, the same analysis suggests that more than half (17) of candidate IEGs also participate in common pathways with known IEGs, involving a densely inter-connected network of 83 significantly over-represented pathways (electronic supplementary material, table S6), including signalling cascades known to mediate the IER, such as the Ca^2+^-dependent pathways and the mitogen-activated protein (MAP) kinase network [29,30].

**Figure 5.**
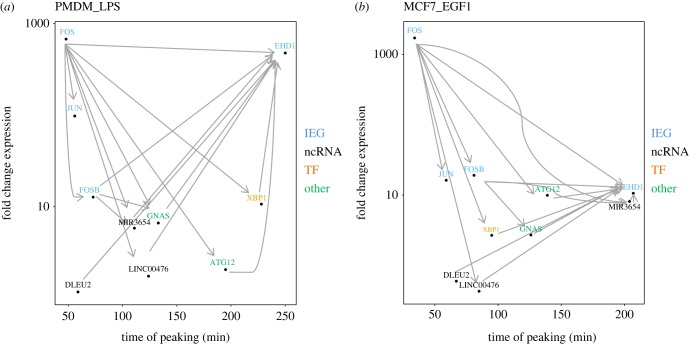
Transcriptional dynamics of genes classified to the peak model. Scatterplots of log fold change against the time of peaking for selected genes, with conserved temporal precedence indicated by arrows for (*a*) PMDM_LPS and (*b*) MCF7_EGF1. FOS peaks earliest and has many conserved temporal relations to later peaking genes, while EHD1 peaks late and has many conserved temporal orderings with earlier peaking genes.

The dynamics of the expression of peak-classified genes can be visualized by a scatterplot of fold change against peak time (electronic supplementary material, figure S7). These quantitative features along with the conserved temporal orderings described above show FOS as the earliest peaking IEG, EHD1 as the last, with an array of conserved orderings subsequent to, and prior to the peaking of these genes, respectively (selected genes plotted in figure 5). The TSSs of known IEGs CAGE tend to show the greatest fold changes (electronic supplementary material, figure S8*a*; Wilcoxon *p* < 2.2 × 10^−16^); however, some candidate IEGs promoters show notably similar timing (electronic supplementary material, figure S8*c*; Wilcoxon *p* = 0.89). The time of peaking is significantly earlier for known IEGs relative to the other protein-coding promoters in only three time series: PMDM_LPS, MCF7_EGF1 and PEC_VEGF. Fold changes in peak ncRNA promoters tend to be lower than for known IEGs (electronic supplementary material, figure S8*b*; Wilcoxon *p* < 0.05), but they occur earlier than known IEGs (electronic supplementary material, figure S8*d*, Wilcoxon *p* < 0.05 for all datasets).

Among the candidate IEGs in the robust set, XBP1 is especially noteworthy. This gene encodes a TF and is relatively short in length (6 kb compared with the mean of 58 kb for all Ensembl protein-coding genes), consistent with the IEG archetype [1]. XBP1 is a highly conserved component of the unfolded protein response (UPR) signalling pathways, activated by unconventional splicing upon endoplasmic reticulum (ER) stress or non-classical anticipatory activation [31–33], and regulates a diverse array of genes involved in ER homeostasis, adipogenesis, lipogenesis and cell survival [34,35]. Interestingly, genes in the robust set are significantly enriched for the GO term GO:003497 *response to ER stress* (FDR < 0.05, all tested genes as the background), and four of the five genes in the robust set sharing this term peak in conserved order across the datasets. Furthermore, we found a significant enrichment (FDR < 0.05) of the XBP1 binding motif in the promoter regions (see Material and methods) of the robust set of genes (electronic supplementary material, figure S9).

## 3. Discussion

Exploiting the precision of FANTOM5 CAGE times series data, we discover a robust set of 42 protein-coding genes driven by promoters showing rapid and transient activation in response to multiple stimuli. This set contains 13 previously known IEGs and 29 candidate IEGs, which are likely to be core components of the IER. Applying our approach to the CAGE TSSs of ncRNAs, we also discovered a set of 15 ncRNAs peaking across at least seven datasets, comprising miRNAs and lncRNAs, suggesting regulatory roles for particular miRNAs and lncRNAs species in the IER [7].

FOS expression has long been considered to lead the IER after cell stimulation [36,37]. Our results on the IER conserved activation network support this, but also similarly conserved relationships extending to an additional 39 coding and non-coding genes. Furthermore, we observed many known and novel IEGs in this network known to be involved in a range of signalling pathways active in the IER, such as the MAPK and the EGF/EGFR signalling pathways. This suggests the variable constellations of genes involved in the IER to any particular stimulus may be underpinned by a deeper level of conservation in the regulation of the IER across stimuli.

One of the most interesting candidate IEGs, XBP1, can be rapidly activated by alternative splicing minutes after cell stimulation with mitogenic hormones, activating peptides such as LPS and cytokines [31–33]. This key event of the induced UPR pathway is a conserved eukaryotic response to cellular stress, and is thought to cooperate in the regulation of IEG expression [32]. However, the dynamics of XBP1 promoter induction in the context of the IER have not been studied previously. Interestingly, we found a significant enrichment for XBP1 TF binding sites in the promoter regions of 11 genes in the IER conserved activated network. The presence of XBP1 and XBP1-responding genes in this network suggests this gene may act as an important link between the IER and the UPR pathway.

## 4. Material and methods

### 4.1. Datasets

The eight datasets used (figure 1) are the most densely sampled human time series produced by the FANTOM5 Project, with all time points represented by three replicates [38]. Detailed information on the generation of these datasets is available from Arner *et al*. [10], including CAGE library preparation, quality control, sequencing and qRT–PCR validation, as well as protocols for CAGE read clustering and TSS detection. All CAGE clusters representing TSSs of protein-coding genes were conservatively thresholded to more than 10 TPM (tags per million), while CAGE clusters corresponding to ncRNA were thresholded to greater than 2 TPM, allowing for their generally lower expression levels. FANTOM5 data downloads, browsers and genomic tools are available from the project website (http://fantom.gsc.riken.jp/5/).

### 4.2. Model-based classification of transcription start site expression profiles

To classify time series data for each CAGE-defined TSS, we refined a previously published method [7] which fits different mathematical models (kinetic signatures) to individual expression profiles, assessing the best fit using nested sampling [39] to compute the marginal likelihood, log *Z*. All time series were normalized such that the medium minimum and maximum across the time series was set to 0 and 10, respectively.

The kinetic signatures considered are: linear, decay, dip and delayed peak (electronic supplementary material, figure S1). The peak kinetic signature considered in the previous method was modified to allow a delay before expression starts to increase in exponential fashion (*t*_d_). Parameter *t*_s_ is the time duration of the initial increase in expression, *p*_1_ is the expression at time 0, and *p*_2_ is the increase in expression at the time of peaking, *t*_p_ = *t*_d_ + *t*_s_.4.1
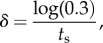
4.2
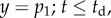
4.3
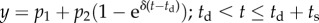
4.4
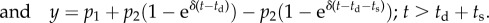
However, an alternative rate 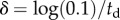 was also used to model the slower dynamics of transcripts peaking later in time, and the best fitting model selected during the decision step. Normalizing the data such that expression lies in the range 0–10 allowed the prior ranges of parameters to be restricted to plausible values that applied to all time series. The fit of models to data was improved as a result. To account for any impact on the log Z calculation, we generated synthetic time series datasets using parameter values drawn at random from the prior ranges to generate one replicate, and generated two other replicates by adding and subtracting (respectively) a given amount of noise to the first. Model fitting was applied to 1000 such datasets per model (using the same noise values for each model on each of the 1000 iterations) and we observed an advantage for each of the more complex models in comparison with the linear model that was consistent over the range of log Z values obtained for the linear model. To offset this effect for each complex model, the advantage (mean difference plus two standard deviations observed in synthetic data) was subtracted from the log Z values calculated for CAGE TSS data when making the categorization decision.

### 4.3. Transcription factor binding site identification

We assessed the enrichment of transcription factor binding site (TFBS) motifs in the JASPAR database [40] (January 2017 release) for all CAGE TSS assigned to genes in the robust set relative to those assigned to the 12 132 genes tested across all the datasets. Motif matches (FDR ≤ 0.05) were sought in flanking 400 bp windows centred on the middle of each CAGE TSS analysed), using FIMO [41] from the MEME package (v. 4.11.2 patch 2). Enrichment of each motif in the robust set relative to the total set was assessed with Fisher's exact tests, correcting for multiple testing (FDR ≤ 0.05).

### 4.4. Pathway and gene ontology enrichment

Functional and pathway enrichments were assessed using GOrilla [13] and InnateDB [28], respectively (FDR ≤ 0.05), using the total 12 132 genes analysed across the eight datasets as the background set.

The list of 234 known IEGs [10] was assembled from 20 published human and mouse datasets from the literature; it is expected to contain few false positives but does include a number of IEGs only reported in cells and/or responses not examined in this study. To compute the enrichment of known IEGs in each dataset, we compared the proportion of peaking CAGE TSSs assigned to IEGs with the proportion of peaking CAGE TSSs assigned to candidate IEGs. For the enrichment of known IEGs in each set of shared peaking genes, we compared the proportion of peaking CAGE TSSs assigned to the IEGs shared in each group of shared genes with the proportion of peaking CAGE TSSs assigned to IEGs in the remaining tested genes. The odds ratio and the *p*-value were assigned using Fisher's exact test.

### 4.5. Network conservation

A total of 57 protein-coding and non-coding candidate IEGs (corresponding to known Ensembl genes) were considered for construction of the conserved activation network. For genes with multiple peaking CAGE TSS, we chose the earliest peaking CAGE TSS (smallest *t*_p_) in each dataset, then the relative pairwise order of each gene was computed with respect to all the other genes in the robust set. For example, if in dataset-1, gene-A peaks before gene-B (*t*_p gene-A_ < *t*_p gene-B_), and this order is observed in six or more of the other seven dataset, the temporal precedence is defined to be conserved. Applying this procedure to all 57 coding and non-coding genes of the robust set, we discovered 40 genes temporally connected by 77 conserved relative orderings (figure 4). The significance of the number of temporal connections observed was measured relative to null distribution, constructed by permuting *t*_p_ for all the CAGE TSSs 1 000 000 times; with the proportion of permuted datasets with at least as many conserved orderings as the observed taken as an empirically derived *p*-value. The observed value (77) was observed or exceeded in 4516 out of 1 000 000 permutations, indicating that the number of temporal connections was statistically significant (*p* < 5 × 10^−3^).

## Supplementary Material

Supplementary data file 1

## Supplementary Material

Supporting information

## Supplementary Material

Figure S1. Methodology

## Supplementary Material

Figure S2. Classifications for protein-coding TSSs

## Supplementary Material

Figure S3. Sharing of peaking TSSs for known IEGs and candidate IEGs in the robust set

## Supplementary Material

Figure S4. IEGs are enriched in genes classified as peaks

## Supplementary Material

Figure S5. Classifications for non-coding RNA TSS

## Supplementary Material

Figure S6. Sharing of peaking TSSs for ncRNA in the robust set

## Supplementary Material

Figure S7

## Supplementary Material

Figure S8. Distributions of expression change and t_p_ across datasets

## Supplementary Material

Figure S9. The regulatory network of the candidate IEG XBP1

## Data accessibility

This article has no additional data.

## References

[1] Fowler T, Sen R, Roy AL 2011 Regulation of primary response genes. Mol. Cell 44, 348–360. (10.1016/j.molcel.2011.09.014) 22055182 PMC3212756

[2] Healy S, Khan P, Davie JR 2013 Immediate early response genes and cell transformation. Pharmacol. Ther. 137, 64–77. (10.1016/j.pharmthera.2012.09.001) 22983151

[3] Greenberg ME, Ziff EB 1984 Stimulation of 3T3 cells induces transcription of the c-fos proto-oncogene. Nature 311, 433–438. (10.1038/311433a0) 6090941

[4] Bahrami S, Drabløs F 2016 Gene regulation in the immediate-early response process. Adv. Biol. Regul. 62, 37–49. (10.1016/j.jbior.2016.05.001) 27220739

[5] O'Donnell A, Odrowaz Z, Sharrocks AD 2012 Immediate-early gene activation by the MAPK pathways: what do and don't we know? Biochem. Soc. Trans. 40, 58–66. (10.1042/BST20110636) 22260666

[6] Fowler T, Suh H, Buratowski S, Roy AL 2013 Regulation of primary response genes in B cells. J. Biol. Chem. 288, 14 906–14 916. (10.1074/jbc.M113.454355) PMC3663512 23536186

[7] Aitken S et al. 2015 Transcriptional dynamics reveal critical roles for non-coding RNAs in the immediate-early response. PLoS Comput. Biol. 11, e1004217 (10.1371/journal.pcbi.1004217) 25885578 PMC4401570

[8] Andersson R et al. 2014 An atlas of active enhancers across human cell types and tissues. Nature 507, 455–461. (10.1038/nature12787) 24670763 PMC5215096

[9] Bar-Joseph Z, Gitter A, Simon I 2012 Studying and modelling dynamic biological processes using time-series gene expression data. Nat. Rev. Genet. 13, 552–564. (10.1038/nrg3244) 22805708

[10] Arner E et al. 2015 Transcribed enhancers lead waves of coordinated transcription in transitioning mammalian cells. Science 347, 1010–1014. (10.1126/science.1259418) 25678556 PMC4681433

[11] Hubbard T et al. 2002 The Ensembl genome database project. Nucleic Acids Res. 30, 38–41. (10.1093/nar/30.1.38) 11752248 PMC99161

[12] Supek F, Bošnjak M, Škunca N, Šmuc T 2011 REVIGO summarizes and visualizes long lists of gene ontology terms. PLoS ONE 6, e21800 (10.1371/journal.pone.0021800) 21789182 PMC3138752

[13] Eden E, Navon R, Steinfeld I, Lipson D, Yakhini Z 2009 GOrilla: a tool for discovery and visualization of enriched GO terms in ranked gene lists. BMC Bioinformatics 10, 1 (10.1186/1471-2105-10-48) 19192299 PMC2644678

[14] Kalam H, Fontana MF, Kumar D 2017 Alternate splicing of transcripts shape macrophage response to Mycobacterium tuberculosis infection. PLoS Pathog. 13, e1006236 (10.1371/journal.ppat.1006236) 28257432 PMC5352146

[15] Stelzer G et al. 2016 The GeneCards suite: from Gene data mining to disease genome sequence analyses. Curr. Protoc. Bioinformatics 54, 1–30. 27322403 10.1002/cpbi.5

[16] Avraham R et al. 2010 EGF decreases the abundance of microRNAs that restrain oncogenic transcription factors. Sci. Signal. 3, ra43 (10.1126/scisignal.2000876) 20516477

[17] Llorens F et al. 2013 Microarray and deep sequencing cross-platform analysis of the mirRNome and isomiR variation in response to epidermal growth factor. BMC Genomics 14, 371 (10.1186/1471-2164-14-371) 23724959 PMC3680220

[18] Liu X et al. 2015 MicroRNA-29a inhibits cell migration and invasion via targeting Roundabout homolog 1 in gastric cancer cells. Mol. Med. Rep. 12, 3944–3950. (10.3892/mmr.2015.3817) 25997819

[19] Zhang Y, Zhou S 2015 MicroRNA 29a inhibits mesenchymal stem cell viability and proliferation by targeting Roundabout 1. Mol. Med. Rep. 12, 6178–6184. (10.3892/mmr.2015.4183) 26252416

[20] Cimmino A et al. 2005 miR-15 and miR-16 induce apoptosis by targeting BCL2. Proc. Natl Acad. Sci. USA 102, 13 944–13 949. (10.1073/pnas.0506654102) PMC1236577 16166262

[21] Lerner M et al. 2009 DLEU2, frequently deleted in malignancy, functions as a critical host gene of the cell cycle inhibitory microRNAs miR-15a and miR-16-1. Exp. Cell Res. 315, 2941–2952. (10.1016/j.yexcr.2009.07.001) 19591824

[22] Gao S-M, Xing C-Y, Chen C-Q, Lin S-S, Dong P-H, Yu F-J 2011 miR-15a and miR-16-1 inhibit the proliferation of leukemic cells by down-regulating WT1 protein level. J. Exp. Clin. Cancer Res. 30, 1 (10.1186/1756-9966-30-1) 22133358 PMC3245444

[23] Gökmen-Polar Y, Zavodszky M, Chen X, Gu X, Kodira C, Badve S 2016 Abstract P2-06-05: LINC00478: a novel tumor suppressor in breast cancer. Cancer Res. 76(4 Suppl.), P2-06-5-P2-5 (10.1158/1538-7445.SABCS15-P2-06-05)

[24] Mills JD, Kavanagh T, Kim WS, Chen BJ, Waters PD, Halliday GM, Janitz M 2015 High expression of long intervening non-coding RNA OLMALINC in the human cortical white matter is associated with regulation of oligodendrocyte maturation. Mol. Brain 8, 1 (10.1186/s13041-014-0091-9) 25575711 PMC4302521

[25] Müller S et al. 2015 Next-generation sequencing reveals novel differentially regulated mRNAs, lncRNAs, miRNAs, sdRNAs and a piRNA in pancreatic cancer. Mol. Cancer 14, 1 (10.1186/1476-4598-14-1) 25910082 PMC4417536

[26] Marín-Béjar O et al. 2013 Pint lincRNA connects the p53 pathway with epigenetic silencing by the Polycomb repressive complex 2. Genome Biol. 14, 1 (10.1186/gb-2013-14-9-r104) PMC4053822 24070194

[27] Wang L, Han S, Jin G, Zhou X, Li M, Ying X, Wang L, Wu H, Zhu Q 2014 Linc00963: a novel, long non-coding RNA involved in the transition of prostate cancer from androgen-dependence to androgen-independence. Int. J. Oncol. 44, 2041–2049. (10.3892/ijo.2014.2363) 24691949

[28] Breuer K et al. 2012 InnateDB: systems biology of innate immunity and beyond—recent updates and continuing curation. Nucleic Acids Res. 41, D1228–D1233. (10.1093/nar/gks1147) 23180781 PMC3531080

[29] Schratt G, Weinhold B, Lundberg AS, Schuck S, Berger J, Schwarz H, Weinberg RA, Ruther U, Nordheim A 2001 Serum response factor is required for immediate-early gene activation yet is dispensable for proliferation of embryonic stem cells. Mol. Cell. Biol. 21, 2933–2943. (10.1128/MCB.21.8.2933-2943.2001) 11283270 PMC86921

[30] Treisman R 1996 Regulation of transcription by MAP kinase cascades. Curr. Opin. Cell Biol. 8, 205–215. (10.1016/S0955-0674(96)80067-6) 8791420

[31] Andruska N, Zheng X, Yang X, Helferich WG, Shapiro DJ 2015 Anticipatory estrogen activation of the unfolded protein response is linked to cell proliferation and poor survival in estrogen receptor *α* positive breast cancer. Oncogene 34, 3760 (10.1038/onc.2014.292) 25263449 PMC4377305

[32] Shapiro DJ, Livezey M, Yu L, Zheng X, Andruska N 2016 Anticipatory UPR activation: a protective pathway and target in cancer. Trends Endocrinol. Metab. 27, 731–741. (10.1016/j.tem.2016.06.002) 27354311 PMC5035594

[33] Skalet AH, Isler JA, King LB, Harding HP, Ron D, Monroe JG 2005 Rapid B cell receptor-induced unfolded protein response in nonsecretory B cells correlates with pro-versus antiapoptotic cell fate. J. Biol. Chem. 280, 39 762–39 771. (10.1074/jbc.M502640200) 16188879

[34] He Y, Sun S, Sha H, Liu Z, Yang L, Xue Z, Chen H, Qi L 2010 Emerging roles for XBP1, a sUPeR transcription factor. Gene Expr. 15, 13–25. (10.3727/105221610X12819686555051) 21061914 PMC3374844

[35] Piperi C, Adamopoulos C, Papavassiliou AG 2016 XBP1: a pivotal transcriptional regulator of glucose and lipid metabolism. Trends Endocrinol. Metab. 27, 119–122. (10.1016/j.tem.2016.01.001) 26803729

[36] Hu E, Mueller E, Oliviero S, Papaioannou V, Johnson R, Spiegelman B 1994 Targeted disruption of the c-fos gene demonstrates c-fos-dependent and-independent pathways for gene expression stimulated by growth factors or oncogenes. EMBO J. 13, 3094. 8039503 10.1002/j.1460-2075.1994.tb06608.x PMC395200

[37] Fei J, Viedt C, Soto U, Elsing C, Jahn L, Kreuzer J 2000 Endothelin-1 and smooth muscle cells. Arterioscler. Thromb. Vasc. Biol. 20, 1244–1249. (10.1161/01.ATV.20.5.1244) 10807739

[38] Lizio M et al. 2015 Gateways to the FANTOM5 promoter level mammalian expression atlas. Genome Biol. 16, 22 (10.1186/s13059-014-0560-6) 25723102 PMC4310165

[39] Aitken S, Akman OE 2013 Nested sampling for parameter inference in systems biology: application to an exemplar circadian model. BMC Syst. Biol. 7, 72 (10.1186/1752-0509-7-72) 23899119 PMC3735395

[40] Mathelier A 2013 JASPAR 2014: an extensively expanded and updated open-access database of transcription factor binding profiles. Nucleic Acids Res. 42, D142–D147. (10.1093/nar/gkt997) 24194598 PMC3965086

[41] Grant CE, Bailey TL, Noble WS 2011 FIMO: scanning for occurrences of a given motif. Bioinformatics 27, 1017–1018. (10.1093/bioinformatics/btr064) 21330290 PMC3065696

