# Supplementary material for: Conserved temporal ordering of promoter activation implicates common mechanisms governing the immediate early response across cell types and stimuli
Source: Open Biol. 2018 Aug 8;8(8):180011. doi: 10.1098/rsob.180011 (PMC6119861; doi:10.1098/rsob.180011)
Supplement: Supporting information [file rsob180011supp2.docx]

# Conserved temporal ordering of promoter activation implicates common mechanisms governing the immediate early response across cell types and stimuli

Annalaura Vacca, Masayoshi Itoh, Hideya Kawaji, Erik Arner, Timo Lassmann, Carsten O. Daub, Piero Carninci, Alistair R. R. Forrest, Yoshihide Hayashizaki, the FANTOM Consortium, Stuart Aitken and Colin A. Semple

# Supporting information

Figure S1. Methodology. For each time series, the Bayesian evidence (log Z) is calculated for each of four predefined models. The time series is classified to the best fitting model. In this example, a time course for the immediate early gene FOS is assigned to the peak model. The prediction of the peak model for the inferred parameters is shown by the blue line in the bottom plot

Figure S2. Classifications for protein-coding TSSs. Stacked barcharts show, for each dataset, the numbers of protein-coding TSSs classified as peak, decay, dip, and linear, and the total percentage of TSS that could be classified.

Figure S3. Sharing of peaking TSSs for known IEGs and candidate IEGs in the robust set . For each gene in the robust set, bar charts show the number of datasets where each TSS is found to peak.

Figure S4. IEGs are enriched in genes classified as peaks. (A) Stacked bars show the numbers of known IEGs (light blue) and TFs (yellow) recovered for the four models of interest (linear, dip, decay and peak) in each time series dataset. Significant enrichment for known IEGs is denoted by an asterisk. Pie charts show the percentage of IEGs contained in the robust set (B) and in the group of genes peaking exclusively in one dataset (C), respectively.

Figure S5. Classifications for non-coding RNA TSS. Stacked barcharts show, for each dataset, the numbers of TSSs for non-coding genes classified as peak, decay, dip, and linear, and the total percentage of TSS that could be classified.

Figure S6. Sharing of peaking TSSs for ncRNA in the robust set . For each ncRNA in the robust set, bar charts show the number of datasets where each TSS is found to peak.

Figure S7. Scatterplots of log fold change against the time of peaking TSS in the robust set for PMDM_LPS (A) and MCF7_EGF1 (B).

Figure S8. Distributions of expression change and t_p_ across datasets. The differences between maximal expression and basal expression are greater for IEG than for other protein coding (A) and for non-coding genes (B). Times of peaking are comparable between peaking IEGs and other protein coding genes (C) but are significantly different between IEGs and non-coding RNAs (significant difference is indicated by an asterisk).

Figure S9. The regulatory network of the candidate IEG XBP1. Conserved temporal precedence between IEGs (light blue nodes) and other protein-coding genes (green nodes) is shown by directed edges. Genes annotated with the GO term ‘response to endoplasmic reticulum stress’ (GO:003497) have a red rectangle around the gene name; red squares indicate genes with CAGE clusters enriched for XBP1 transcription factor binding sites.

**File S1. Peaking times for genes in the robust set in all dataset.** Genes and TSS coordinates together with peaking times and classification of genes as IEGs or transcription factors.

Table S1. Datasets interrogated.

|  | **PMDM_LPS**  **(Protein coding/ non coding)** | **PAC_FGF2**  **(Protein coding/ non coding)** | **PAC_IL1B**  **(Protein coding/ non coding)** | **MCF7_HRG**  **(Protein coding/ non coding)** |
| --- | --- | --- | --- | --- |
| **# CAGE clusters** | 14,376 / 1,209 | 11,235 / 1,202 | 10,513 / 1,210 | 11,513 / 1,342 |
| **# Encode genes** | 8,951 / 673 | 8,112 / 692 | 7,706 / 681 | 8,511 / 763 |
|  | **MCF7_EGF1**  **(Protein coding/ non coding)** | **PEC_VEGF**  **(Protein coding/ non coding)** | **PMSC_MIX**  **(Protein coding/ non coding)** | **SAOS2_OST**  **(Protein coding/ non coding)** |
| **# CAGE clusters** | 11,352 / 1,345 | 10,686 / 1,377 | 13,881 / 1,252 | 11,521 / 1,640 |
| **# Encode genes** | 8,458 / 769 | 8,040 / 772 | 9,197 / 692 | 8,639 / 858 |

The table summarizes the number of CAGE clusters (representing TSSs) and Ensembl genes used as input for each dataset.

Table S2. Classification of promoter dynamics.

|  | **PMDM_LPS** | | **PAC_FGF2** | | **PAC_IL1B** | | **MCF7_HRG** | |
| --- | --- | --- | --- | --- | --- | --- | --- | --- |
|  | **#CAGE clusters** | **#genes** | **#CAGE clusters** | **#genes** | **#CAGE clusters** | **#genes** | **#CAGE clusters** | **#genes** |
| **No decision** | 8382 | 5890 | 7601 | 5894 | 6666 | 5356 | 7559 | 6045 |
| **Peak** | 3689 | 2731 | 3225 | 2852 | 3483 | 3053 | 2527 | 2200 |
| **Decay** | 1114 | 998 | 34 | 34 | 41 | 41 | 415 | 400 |
| **Dip** | 608 | 585 | 368 | 360 | 313 | 306 | 779 | 740 |
| **Linear** | 583 | 541 | 7 | 7 | 10 | 10 | 233 | 231 |
|  | **MCF7_EGF1** | | **PEC_VEGF** | | **PMSC_MIX** | | **SAOS2_OST** | |
|  | **#CAGE clusters** | **#genes** | **#CAGE clusters** | **#genes** | **#CAGE clusters** | **#genes** | **#CAGE clusters** | **#genes** |
| **No decision** | 7370 | 5906 | 8072 | 6469 | 11793 | 8228 | 8421 | 6731 |
| **Peak** | 2771 | 2424 | 2182 | 1941 | 1836 | 1672 | 2330 | 2105 |
| **Decay** | 119 | 115 | 46 | 45 | 71 | 67 | 235 | 226 |
| **Dip** | 967 | 904 | 354 | 353 | 101 | 100 | 486 | 470 |
| **Linear** | 125 | 123 | 32 | 32 | 80 | 79 | 49 | 49 |

Number of protein coding CAGE clusters and Ensembl genes classified to each model (or to no models: ‘No decision’) in each dataset.

Table S3. Confusion matrix of the intersection between non-robust peaking genes in the eight datasets.

|  | **PMDM_LPS** | **MCF7_EGF1** | **MCF7_HRG** | **MPSC_MIX** | **PAC_FGF2** | **PAC_IL1B** | **PEC_VEGF** | **SAOS2_OST** |
| --- | --- | --- | --- | --- | --- | --- | --- | --- |
| **PMDM_LPS** | 709 | 72 | 59 | 61 | 129 | 133 | 93 | 92 |
|  | **MCF7_EGF1** | 363 | 190 | 42 | 82 | 106 | 52 | 52 |
|  |  | **MCF7_HRG** | 381 | 41 | 74 | 59 | 37 | 64 |
|  |  |  | **MPSC_MIX** | 456 | 87 | 86 | 37 | 53 |
|  |  |  |  | **PAC_FGF2** | 461 | 231 | 75 | 90 |
|  |  |  |  |  | **PAC_IL1B** | 489 | 111 | 110 |
|  |  |  |  |  |  | **PEC_VEGF** | 291 | 69 |
|  |  |  |  |  |  |  | **SAOS2_OST** | 452 |

Confusion matrix of the number of peaking genes shared by less than 7 datasets (excluding the robust set). The table presents the number of genes shared only by each pair of datasets. As expected the pairs MCF7_HRG - MCF7_EGF1 and PAC_FGF2 – PAC_IL1B are characterized by a greater overlap than the other pairs.

Table S4. IEG enrichment across models and datasets.

| **Dataset** | **Peak** | | | **Dip** | | | | | **Decay** | | |
| --- | --- | --- | --- | --- | --- | --- | --- | --- | --- | --- | --- |
|  | **# IEG clusters/**  **total clusters** | **OR** | **p** | **# IEG clusters/**  **total clusters** | | **OR** | | **p** | **# IEG clusters/**  **total clusters** | **OR** | **p** |
| **PMDM_LPS** | 264/ 3,689 | 5.8 | 2.2e-16 | 2/ 608 | | 0.1 | | 7.3e-6 | 8/ 1,114 | 0.2 | 6.2e-7 |
| **PAC_FGF2** | 87/ 3,225 | 1.5 | 4.2e-3 | 8/ 368 | | 1.1 | | 0.9 | 4/ 34 | 6.4 | 5.1e-3 |
| **PAC_IL1B** | 116/ 3,483 | 2.1 | 9.1e-8 | 3/ 313 | | 0.4 | | 0.2 | 2/ 41 | 2.3 | 0.2 |
| **MCF7_HRG** | 140/ 2,527 | 6.4 | 2.2e-16 | 10/ 779 | | 0.6 | | 0.2 | 7/ 415 | 0.9 | 0.9 |
| **MCF7_EGF1** | 71/ 2,771 | 1.9 | 3.3e-5 | 21/ 967 | | 1.4 | | 0.2 | 2/ 119 | 1 | 0.7 |
| **PEC_VEGF** | 127/ 2,182 | 4.3 | 2.2e-16 | 6/ 354 | | 0.7 | | 0.6 | 1/ 46 | 0.9 | 1 |
| **PMSC_MIX** | 36/ 1,836 | 1.3 | 0.2 | 2/ 101 | | 1.3 | | 0.7 | 16/ 71 | 19.7 | 1.1e-14 |
| **SAOS2_OST** | 72/ 2,330 | 3.6 | 4.0e-14 | 5/ 486 | | 0.8 | | 0.8 | 2/ 235 | 0.6 | 0.8 |
| **Dataset** | **Linear** | | | **No decision** | | | | | **Tot tested** | | |
|  | **# IEG clusters/**  **total clusters** | **OR** | **p** | **# IEG clusters/**  **total clusters** | **OR** | | **p** | | **# IEG clusters/ total clusters** | | |
| **PMDM_LPS** | 1/ 582 | 0.1 | 1.3e-6 | 130/ 8,382 | 0.3 | | 2.2e-16 | | 405/14,376 | | |
| **PAC_FGF2** | 1/ 7 | 7.9 | 0.1 | 133/ 7,601 | 0.6 | | 6.6e-4 | | 233/11,235 | | |
| **PAC_IL1B** | 1/ 10 | 4.9 | 0.2 | 110/ 6,666 | 0.5 | | 5.7e-7 | | 232/10,513 | | |
| **MCF7_HRG** | 1/ 233 | 0.2 | 0.1 | 63/ 7,559 | 0.2 | | 2.2e-16 | | 221/11,513 | | |
| **MCF7_EGF1** | 2/ 125 | 1 | 1 | 91/ 7,370 | 0.5 | | 4.4e-6 | | 187/11,352 | | |
| **PEC_VEGF** | 0/ 32 | 0 | 1 | 114/  8,072 | 0.3 | | 2.2e-16 | | 248/10,686 | | |
| **PMSC_MIX** | 3/ 80 | 2.5 | 0.1 | 160/  11,793 | 0.5 | | 1.4e-5 | | 217/13881 | | |
| **SAOS2_OST** | 0/ 49 | 0 | 1 | 73/ 8,421 | 0.3 | | 6.0e-11 | | 152/11521 | | |

Enrichment was computed using Fisher’s exact test.

Table S5. GO term enrichment analysis.

| **GO term id** | **Description** | **FDR q-value**  **(peaking genes)** | **Enrichment (peaking genes)** |
| --- | --- | --- | --- |
| GO:0044237 | cellular metabolic process | 0.0000000226 | 1.03 |
| GO:0008152 | metabolic process | 0.000000121 | 1.03 |
| GO:0051704 | multi-organism process | 0.00000344 | 1.11 |
| GO:0009892 | negative regulation of metabolic process | 0.0000135 | 1.07 |
| GO:0043620 | regulation of DNA-templated transcription in response to stress | 0.000021 | 1.29 |
| GO:0033554 | cellular response to stress | 0.0000255 | 1.09 |
| GO:0071704 | organic substance metabolic process | 0.0000302 | 1.03 |
| GO:0010605 | negative regulation of macromolecule metabolic process | 0.0000381 | 1.07 |
| GO:0043170 | macromolecule metabolic process | 0.0000451 | 1.03 |
| GO:0006807 | nitrogen compound metabolic process | 0.0000459 | 1.03 |
| GO:0044260 | cellular macromolecule metabolic process | 0.0000497 | 1.03 |
| GO:0043618 | regulation of transcription from RNA polymerase II promoter in response to stress | 0.0000517 | 1.29 |
| GO:0009987 | cellular process | 0.0000883 | 1.01 |
| GO:0051716 | cellular response to stimulus | 0.000227 | 1.06 |
| GO:0010629 | negative regulation of gene expression | 0.000278 | 1.08 |
| GO:0032268 | regulation of cellular protein metabolic process | 0.00045 | 1.06 |
| GO:0042221 | response to chemical | 0.000533 | 1.06 |
| GO:0044238 | primary metabolic process | 0.000538 | 1.02 |
| GO:0010604 | positive regulation of macromolecule metabolic process | 0.000961 | 1.05 |
| GO:0051246 | regulation of protein metabolic process | 0.0011 | 1.06 |
| GO:0007568 | aging | 0.0012 | 1.19 |
| GO:0051726 | regulation of cell cycle | 0.00151 | 1.08 |
| GO:0010628 | positive regulation of gene expression | 0.00164 | 1.07 |
| GO:0009893 | positive regulation of metabolic process | 0.0029 | 1.05 |
| GO:0045862 | positive regulation of proteolysis | 0.00296 | 1.14 |
| GO:0008150 | biological_process | 0.003 | 1.01 |
| GO:0006357 | regulation of transcription from RNA polymerase II promoter | 0.0031 | 1.06 |
| GO:0031324 | negative regulation of cellular metabolic process | 0.00374 | 1.05 |
| GO:0006950 | response to stress | 0.00374 | 1.05 |
| GO:0048585 | negative regulation of response to stimulus | 0.00386 | 1.07 |
| GO:1901698 | response to nitrogen compound | 0.00482 | 1.10 |
| GO:0030162 | regulation of proteolysis | 0.00481 | 1.10 |
| GO:0010033 | response to organic substance | 0.00577 | 1.06 |
| GO:0006979 | response to oxidative stress | 0.00584 | 1.14 |
| GO:0045321 | leukocyte activation | 0.00806 | 1.09 |
| GO:0048522 | positive regulation of cellular process | 0.00814 | 1.03 |
| GO:0042542 | response to hydrogen peroxide | 0.00822 | 1.23 |
| GO:1901360 | organic cyclic compound metabolic process | 0.00834 | 1.03 |
| GO:0023057 | negative regulation of signaling | 0.00871 | 1.07 |
| GO:0009968 | negative regulation of signal transduction | 0.00943 | 1.08 |
| GO:0045787 | positive regulation of cell cycle | 0.00983 | 1.13 |
| GO:0010648 | negative regulation of cell communication | 0.00978 | 1.07 |
| GO:0043900 | regulation of multi-organism process | 0.0104 | 1.13 |
| GO:0051172 | negative regulation of nitrogen compound metabolic process | 0.0109 | 1.05 |
| GO:0010035 | response to inorganic substance | 0.0115 | 1.12 |
| GO:0050896 | response to stimulus | 0.0117 | 1.03 |
| GO:0031325 | positive regulation of cellular metabolic process | 0.012 | 1.04 |
| GO:1904951 | positive regulation of establishment of protein localization | 0.0121 | 1.11 |
| GO:0080134 | regulation of response to stress | 0.0124 | 1.07 |
| GO:2001234 | negative regulation of apoptotic signaling pathway | 0.0124 | 1.17 |
| GO:0010557 | positive regulation of macromolecule biosynthetic process | 0.0126 | 1.06 |
| GO:0043069 | negative regulation of programmed cell death | 0.0145 | 1.09 |
| GO:2001233 | regulation of apoptotic signaling pathway | 0.0156 | 1.12 |
| GO:0051592 | response to calcium ion | 0.0159 | 1.24 |
| GO:1901700 | response to oxygen-containing compound | 0.0163 | 1.07 |
| GO:0043066 | negative regulation of apoptotic process | 0.0165 | 1.09 |
| GO:0016070 | RNA metabolic process | 0.0165 | 1.04 |
| GO:0019222 | regulation of metabolic process | 0.0164 | 1.03 |
| GO:0010243 | response to organonitrogen compound | 0.0164 | 1.09 |
| GO:0010468 | regulation of gene expression | 0.0168 | 1.03 |
| GO:0051173 | positive regulation of nitrogen compound metabolic process | 0.0173 | 1.04 |
| GO:0000302 | response to reactive oxygen species | 0.0176 | 1.18 |
| GO:0006915 | apoptotic process | 0.0176 | 1.10 |
| GO:0043067 | regulation of programmed cell death | 0.0181 | 1.06 |
| GO:0042981 | regulation of apoptotic process | 0.0191 | 1.06 |
| GO:0034599 | cellular response to oxidative stress | 0.0197 | 1.16 |
| GO:0051222 | positive regulation of protein transport | 0.0234 | 1.11 |
| GO:0001775 | cell activation | 0.0235 | 1.08 |
| GO:0006725 | cellular aromatic compound metabolic process | 0.025 | 1.03 |
| GO:0032269 | negative regulation of cellular protein metabolic process | 0.0249 | 1.07 |
| GO:0038061 | NIK/NF-kappaB signaling | 0.0252 | 1.26 |
| GO:0060548 | negative regulation of cell death | 0.0263 | 1.08 |
| GO:0048519 | negative regulation of biological process | 0.0272 | 1.03 |
| GO:0046483 | heterocycle metabolic process | 0.0297 | 1.03 |
| GO:0033138 | positive regulation of peptidyl-serine phosphorylation | 0.0339 | 1.24 |
| GO:0010941 | regulation of cell death | 0.0339 | 1.06 |
| GO:0060255 | regulation of macromolecule metabolic process | 0.0353 | 1.02 |
| GO:0051248 | negative regulation of protein metabolic process | 0.0363 | 1.07 |
| GO:0048518 | positive regulation of biological process | 0.0371 | 1.03 |
| GO:0006139 | nucleobase-containing compound metabolic process | 0.0392 | 1.03 |
| GO:0051247 | positive regulation of protein metabolic process | 0.0394 | 1.05 |
| GO:0043903 | regulation of symbiosis, encompassing mutualism through parasitism | 0.04 | 1.15 |
| GO:2001236 | regulation of extrinsic apoptotic signaling pathway | 0.0401 | 1.17 |
| GO:0007623 | circadian rhythm | 0.0425 | 1.20 |
| GO:0009891 | positive regulation of biosynthetic process | 0.045 | 1.05 |
| GO:0044092 | negative regulation of molecular function | 0.0451 | 1.07 |
| GO:1901576 | organic substance biosynthetic process | 0.047 | 1.03 |
| GO:0032270 | positive regulation of cellular protein metabolic process | 0.0471 | 1.06 |

Significantly enriched GO terms common to peaking genes and IEGs (FDR corrected hypergeometric test q-value <0.05).

Table S6. Biological pathways overrepresentation.

| **Genes** | **Overrepresented pathways (P-value corrected <0.05)** |
| --- | --- |
| **FOS; JUN** | Tsp-1 induced apoptosis in microvascular endothelial cell  Pertussis toxin-insensitive ccr5 signaling in macrophage  Activation of the AP-1 family of transcription factors  S1P2 pathway  Calcium signaling by hbx of hepatitis b virus  Repression of pain sensation by the transcriptional regulator dream  Cadmium induces dna synthesis and proliferation in macrophages  Nerve growth factor pathway (ngf)  Mets affect on macrophage differentiation  Oxidative stress induced gene expression via nrf2  Igf-1 signaling pathway  PDGFR-alpha signaling pathway  Inhibition of cellular proliferation by gleevec  Tpo signaling pathway  IL12 signaling mediated by STAT4  Pdgf signaling pathway  Endothelins  ErbB2/ErbB3 signaling events  Osteopontin-mediated events  Fc epsilon receptor i signaling in mast cells  Bcr signaling pathway  RhoA signaling pathway  Role of egf receptor transactivation by gpcrs in cardiac hypertrophy  MAPK targets/ Nuclear events mediated by MAP kinases  Signal transduction through il1r  Toll-like receptor pathway  Angiotensin ii mediated activation of jnk pathway via pyk2 dependent signaling  IL2-mediated signaling events  IL6-mediated signaling events  LPA receptor mediated events  Presenilin action in Notch and Wnt signaling  FCERI mediated MAPK activation  Keratinocyte differentiation  T cell receptor signaling pathway  Mapkinase signaling pathway  MAP kinase activation in TLR cascade  BCR signaling pathway  Colorectal cancer  Regulation of nuclear SMAD2/3 signaling  Leishmaniasis  B cell receptor signaling pathway |
| **FOS; JUN; DUSP1** | Fc-epsilon receptor I signaling in mast cells |
| **FOS; JUN; FOSL1** | Calcium signaling in the CD4+ TCR pathway  Downstream signaling in naive CD8+ T cells |
| **FOS; JUN; FOSB; FOSL1** | CD4 T cell receptor signaling Osteoclast differentiation |
| **FOS; JUN; FOSB** | BCR |
| **FOS; JUN; SGK1** | Glucocorticoid receptor regulatory network IL6 |
| **DUSP1; JUN** | Mechanism of gene regulation by peroxisome proliferators via ppara |
| **FOSL1; JUN** | Validated transcriptional targets of AP1 family members Fra1 and Fra2 |
| **FOS; FOSL1** | Bone remodeling |
| **FOS; JUN**; PTGES3; DKC1 | Regulation of telomerase |
| **FOS; JUN; DUSP1;** CUL3 | ATF-2 transcription factor network |
| **FOS; JUN; DUSP1**; ARF4 | ErbB1 downstream signalling |
| **FOS; JUN; DUSP1;** FLNA | MAPK signaling pathway |
| **FOS; JUN; BHLHE40;** PFKFB3 | HIF-1-alpha transcription factor network |
| **FOS; JUN**; PLEC; RPSA | Alpha6Beta4Integrin |
| **FOS; JUN**; XBP1 | FOXA1 transcription factor network |
| **FOS; JUN; FOSL1**; SLC3A2 | Calcineurin-regulated NFAT-dependent transcription in lymphocytes |
| **FOS; JUN; SDC4;** | FGF signaling pathway |
| **FOS; JUN;** FLNA | Prolactin |
| **FOS; JUN;** UBE2D3 | MyD88-independent cascade  TRIF-mediated TLR3/TLR4 signaling  Toll Like Receptor 3 (TLR3) Cascade  Activated TLR4 signaling  Toll Like Receptor 4 (TLR4) Cascade  Toll-Like Receptors Cascades |
| **FOS; JUN; FOSB; PPP1R15A;** UBE2D3 | TGF_beta_Receptor |
| **FOS; JUN;** PTGES3; UBE2D3 | Cellular responses to stress |
| **FOS; JUN; SDC4; DUSP1**; ARF4; PLEC | EGFR1 |
| **FOS; JUN;** GNAS | Chagas disease (American trypanosomiasis) |
| **JUN**; B4GALT1 | Pre-NOTCH Expression and Processing |
| **JUN**; GNB2L1 | Regulation of Androgen receptor activity IL5 |
| **JUN; SGK1**; ITM2B | IL2 |
| **JUN**; FLNA; GNB2L1; UBE2D3 | TNFalpha |
| **SDC4**; THBS1 | Syndecan interactions  Syndecan-4-mediated signaling events  Beta3 integrin cell surface interactions  Non-integrin membrane-ECM interactions |
| ATG12; UBE2D3 | Negative regulators of RIG-I/MDA5 signaling |

Pathway analysis of the 42 genes in the robust set. Known IEGs are indicated in bold.
