# Supplementary figures and images for: Conserved temporal ordering of promoter activation implicates common mechanisms governing the immediate early response across cell types and stimuli
Source: Open Biol. 2018 Aug 8;8(8):180011. doi: 10.1098/rsob.180011 (PMC6119861; doi:10.1098/rsob.180011)

## FOS (IEG)

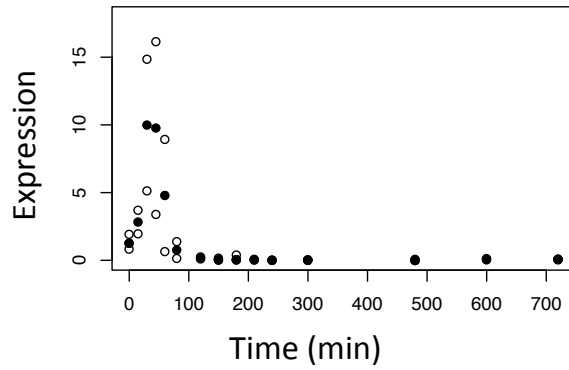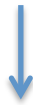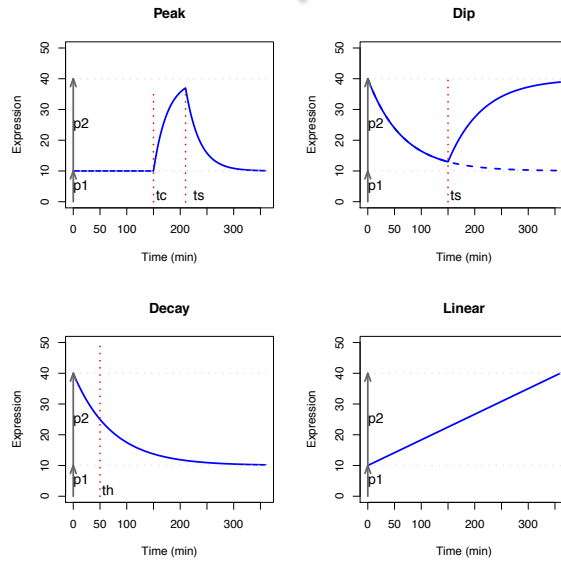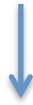

## FOS (IEG)

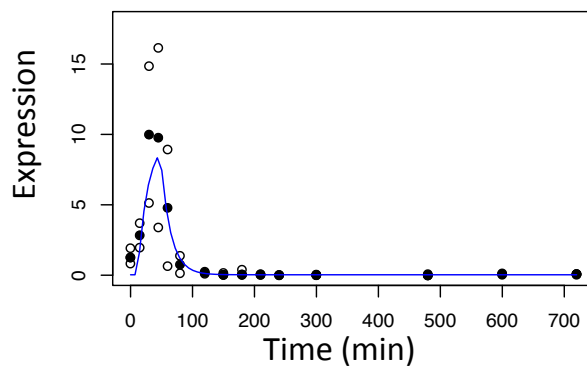

Supplement: Figure S1. Methodology [file rsob180011supp3.pdf]

A

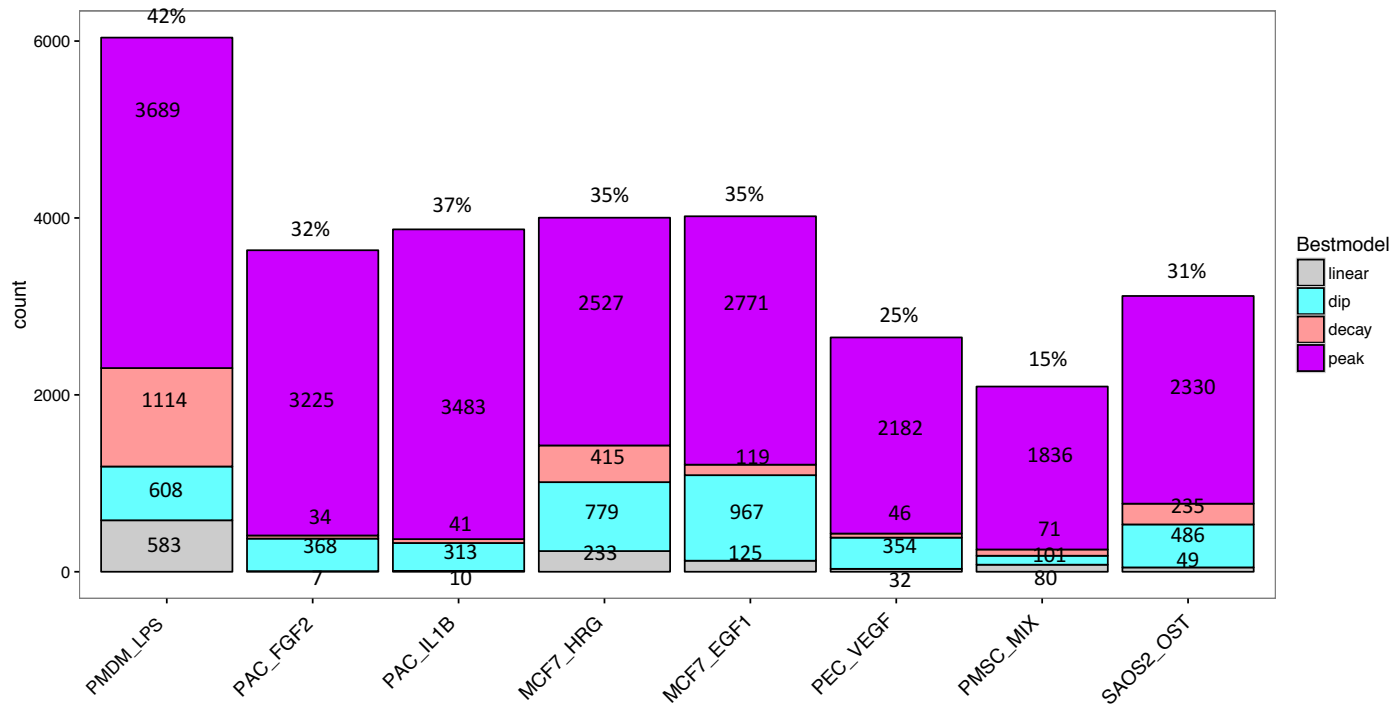

Supplement: Figure S2. Classifications for protein-coding TSSs [file rsob180011supp4.pdf]

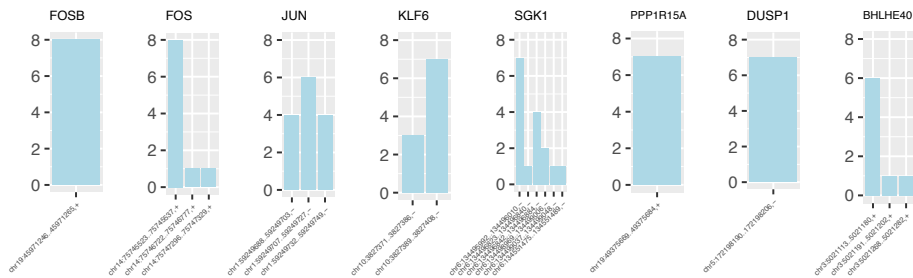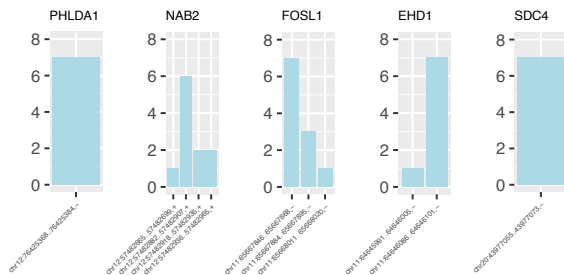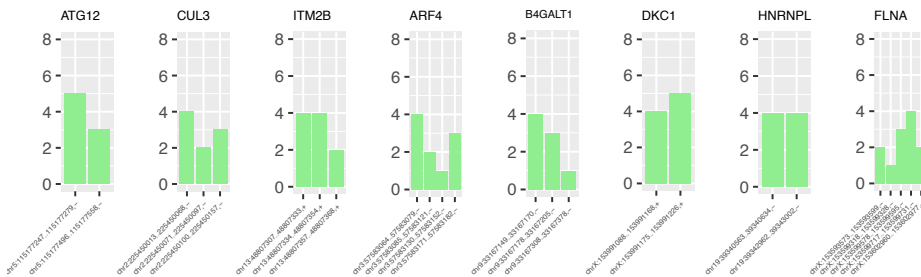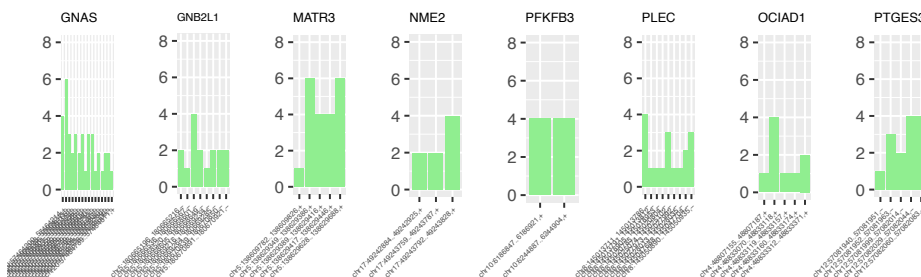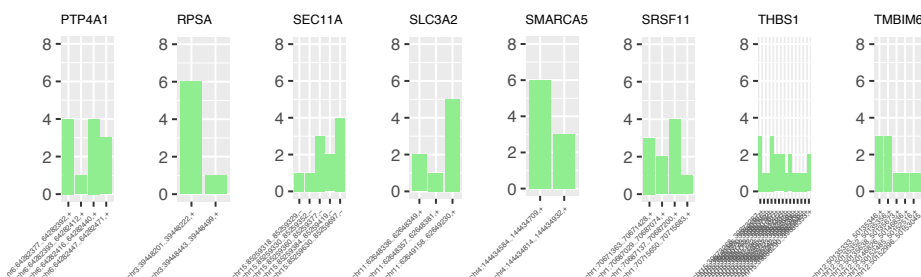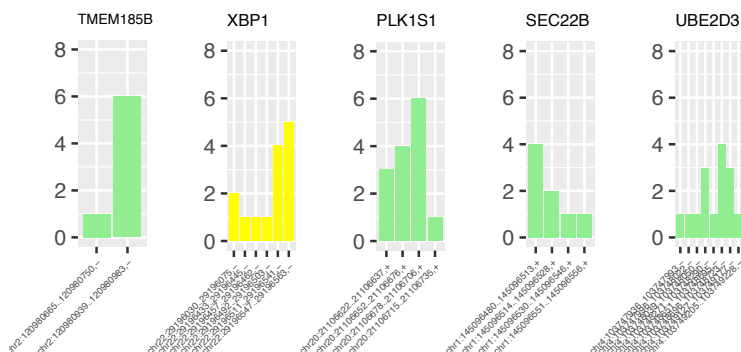

Supplement: Figure S3. Sharing of peaking TSSs for known IEGs and candidate IEGs in the robust set [file rsob180011supp5.pdf]

A

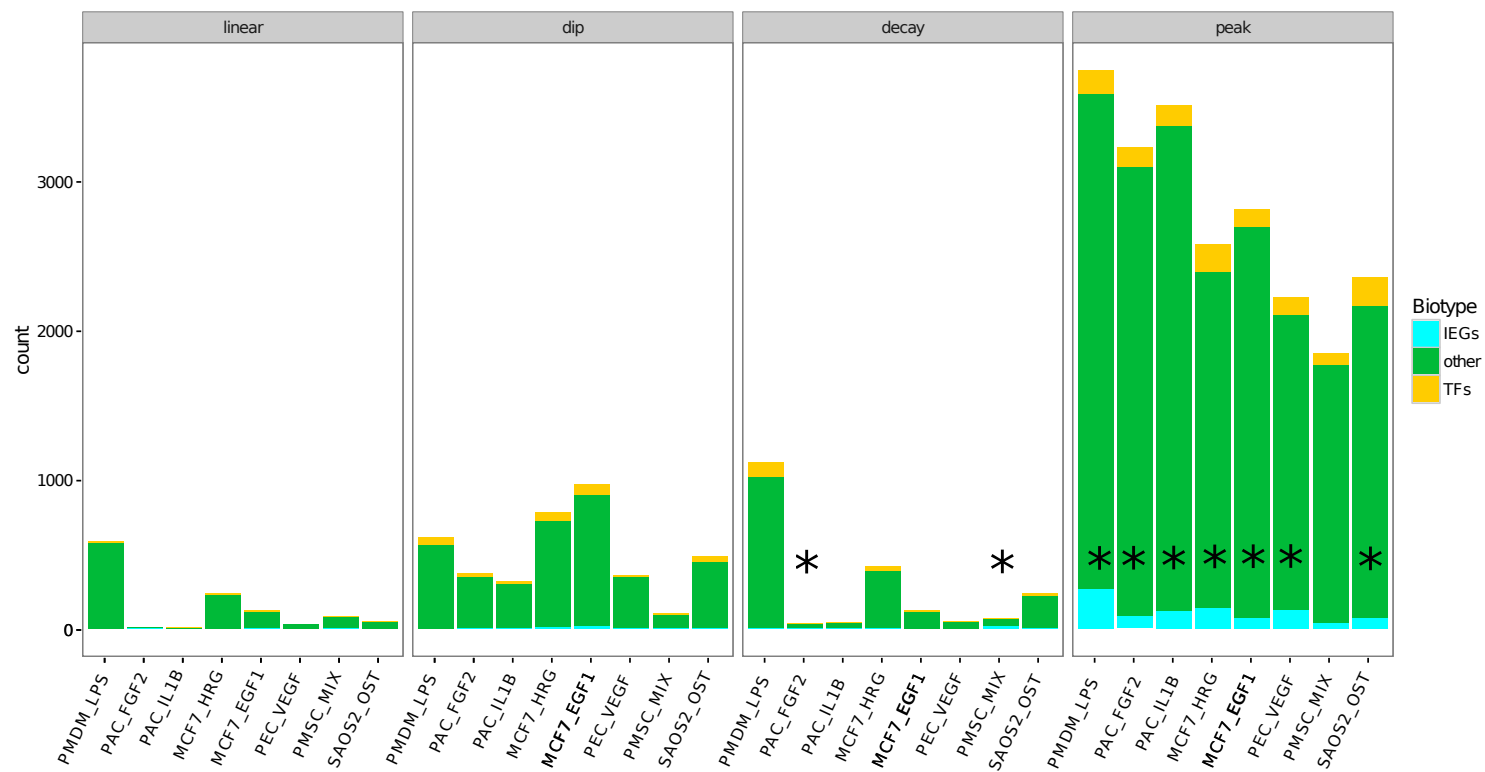

B

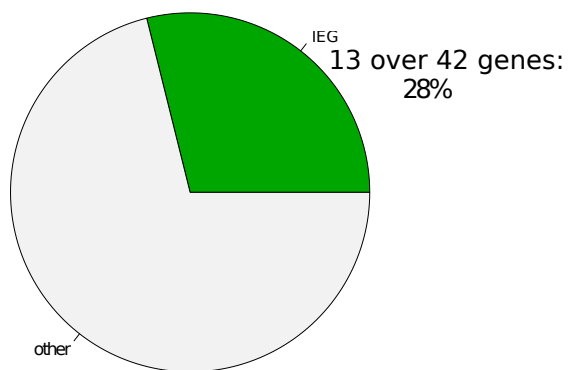

Dataset

C

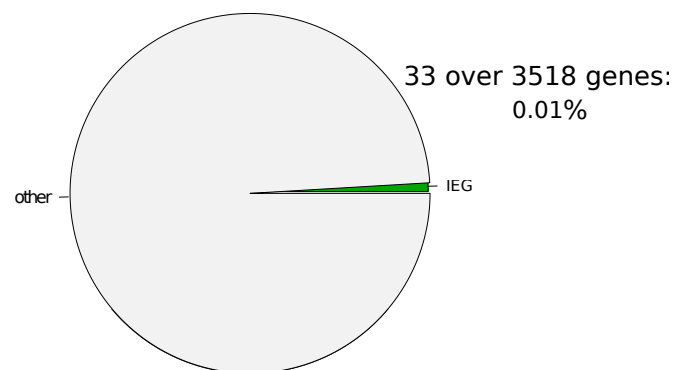

Supplement: Figure S4. IEGs are enriched in genes classified as peaks [file rsob180011supp6.pdf]

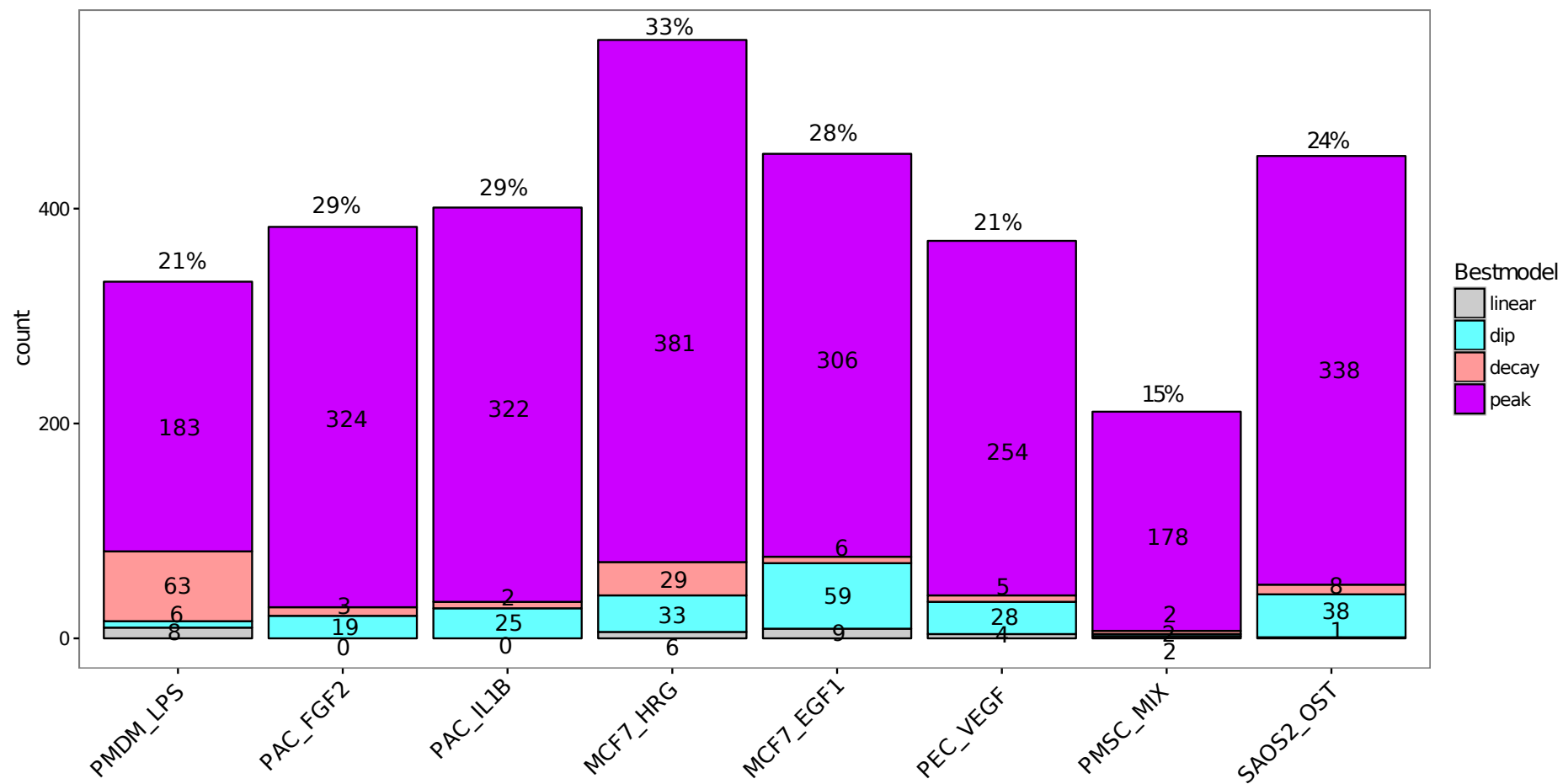

Supplement: Figure S5. Classifications for non-coding RNA TSS [file rsob180011supp7.pdf]

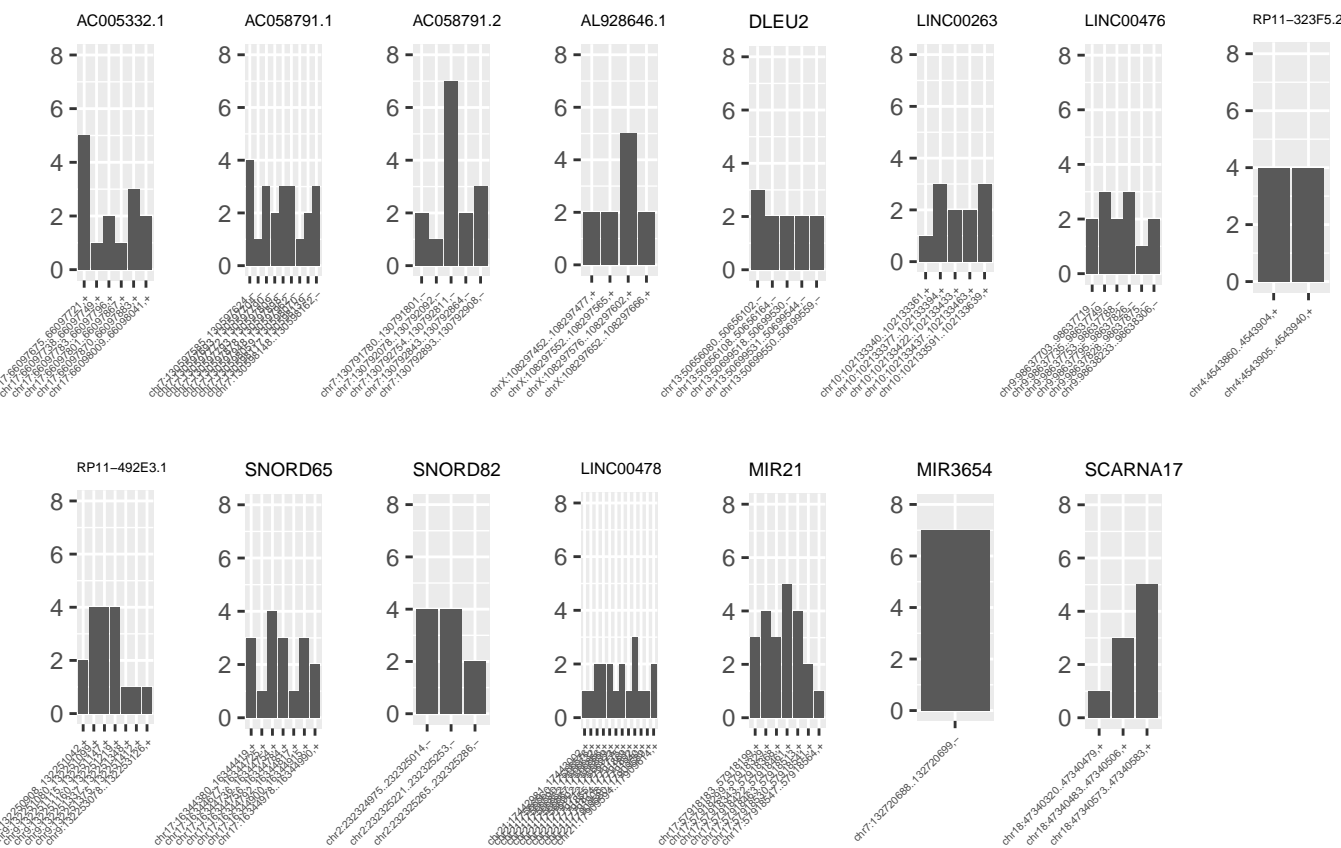

Supplement: Figure S6. Sharing of peaking TSSs for ncRNA in the robust set [file rsob180011supp8.pdf]

(a)

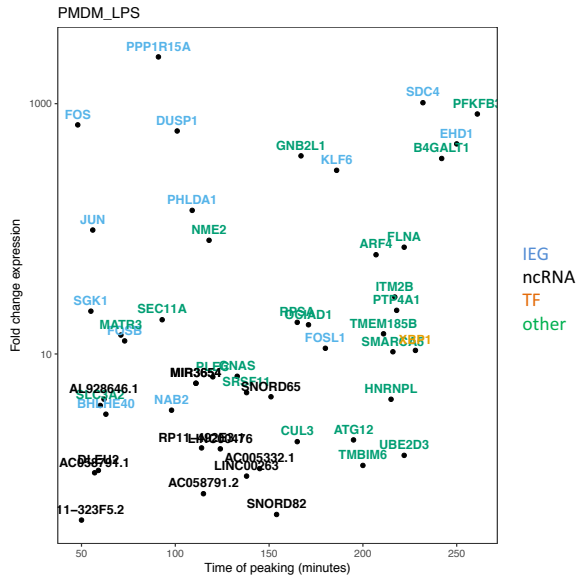

(b)

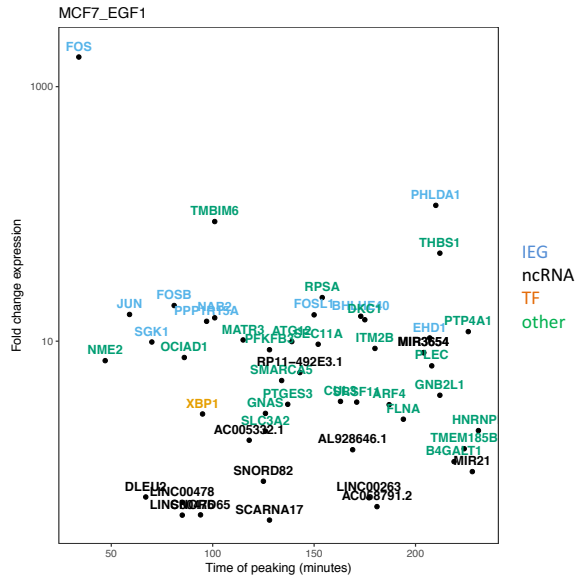

Supplement: Figure S7 [file rsob180011supp9.pdf]

A

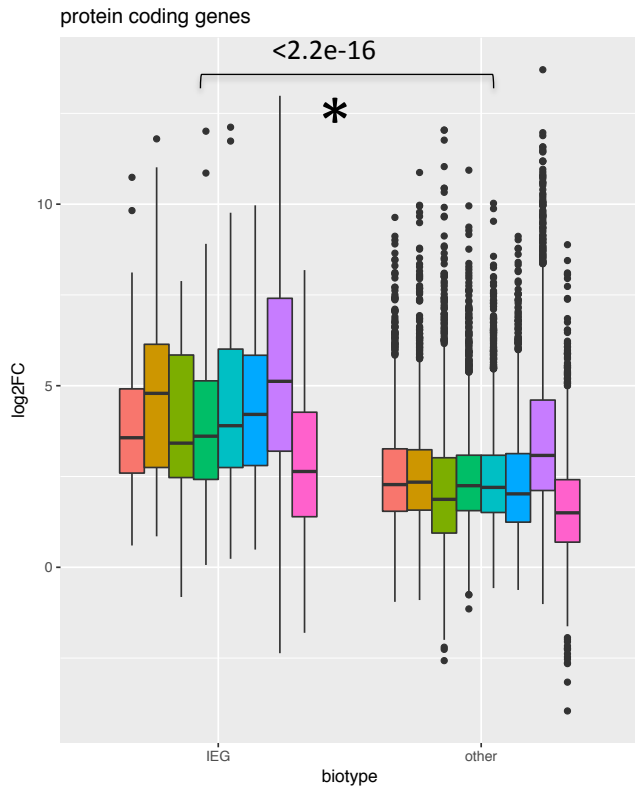

B

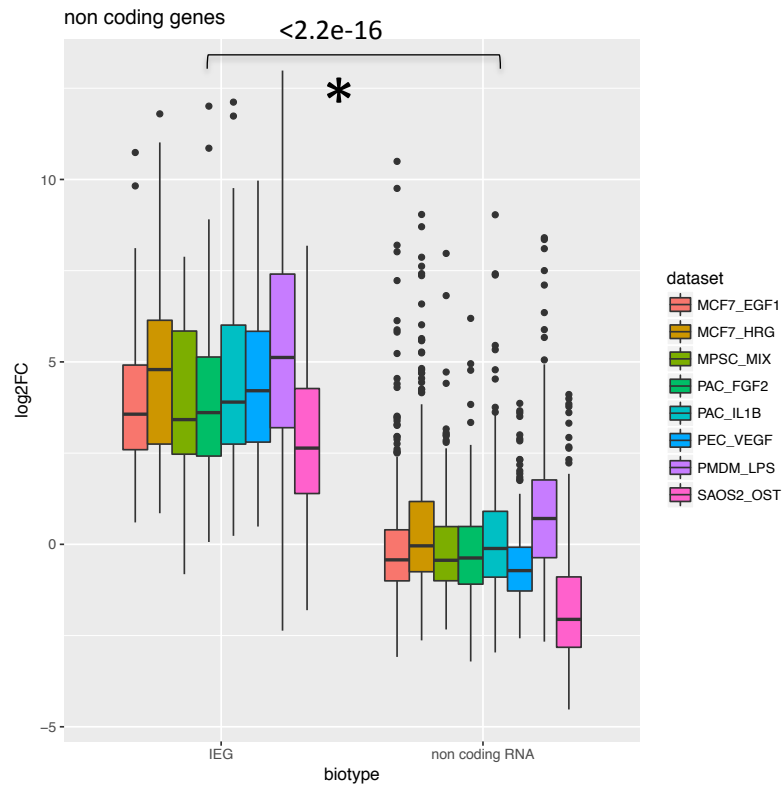

C

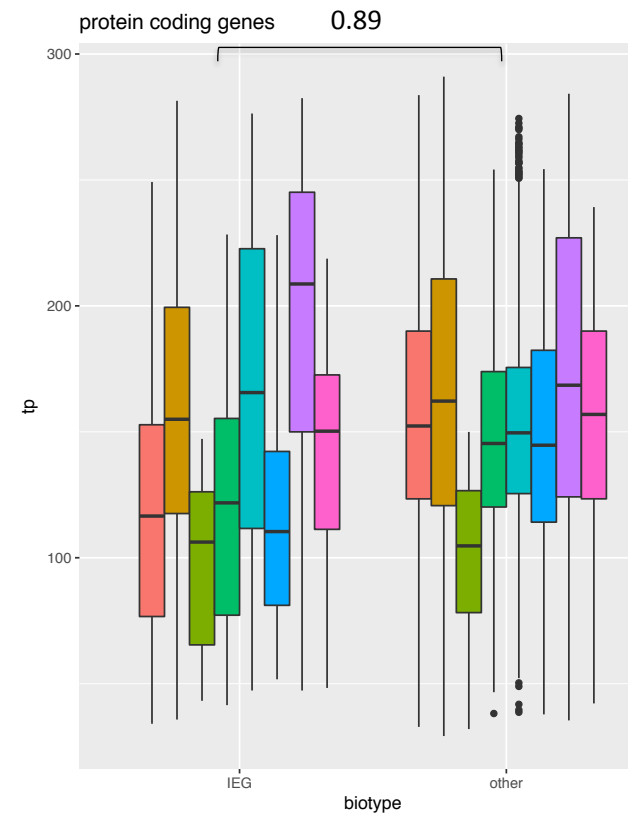

D

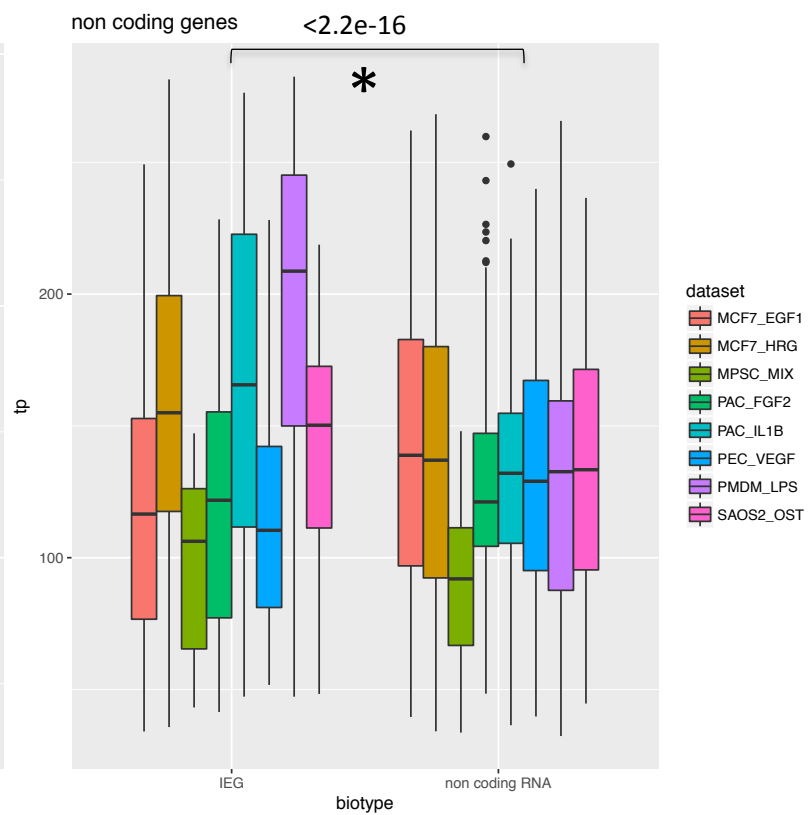

Supplement: Figure S8. Distributions of expression change and tp across datasets [file rsob180011supp10.pdf]

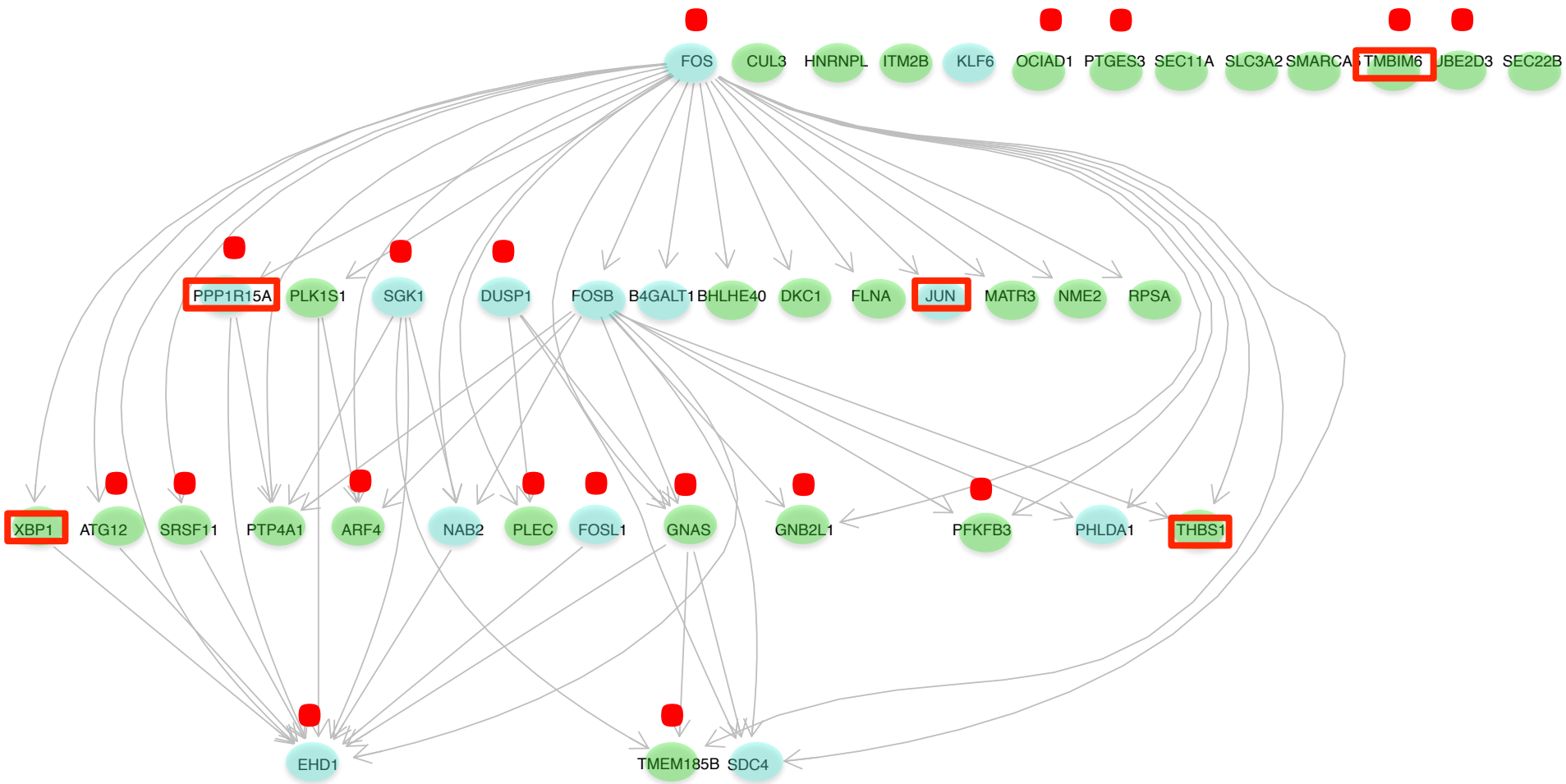

Supplement: Figure S9. The regulatory network of the candidate IEG XBP1 [file rsob180011supp11.pdf]
